# Cell-type-specific localization of huntingtin-associated protein 1 in neuroendocrine and neurosecretory systems of the mouse hypothalamic–pituitary axis

**DOI:** 10.3389/fncel.2026.1963485

**Published:** 2026-10-26

**Authors:** Naoya Horiguchi, Md Nabiul Islam, Yui Kobayashi, Akie Yanai, Shintaro Oka, Koji Shiraishi, Koh Shinoda

**Affiliations:** 1 Department of Urology, Yamaguchi University Graduate School of Medicine, Ube, Minami-Kogushi, Japan; 2 Division of Neuroanatomy, Department of Neuroscience, Yamaguchi University Graduate School of Medicine, Ube, Minami-Kogushi, Japan; 3 Department of Basic Laboratory Sciences, Faculty of Medicine and Health Sciences, Yamaguchi University Graduate School of Medicine, Ube, Minami-Kogushi, Japan; 4 School of Human Care Studies, Nagoya University of Arts and Sciences, Takenoyama, Nisshin, Aichi, Japan

**Keywords:** arginine vasopressin, HAP1, hypothalamic–pituitary axis, neuroendocrine system, oxytocin, pituitary gland, selective cellular vulnerability

## Abstract

Huntingtin-associated protein 1 (HAP1) is a neurocytoplasmic protein implicated in intracellular trafficking, vesicular transport, and neuroprotection. Although HAP1 is abundantly expressed in hypothalamic regions involved in neuroendocrine regulation, its distribution and cellular specificity within the hypothalamic–pituitary axis remain poorly understood. Therefore, the present study aimed to characterize the expression pattern and cellular localization of HAP1 in neuroendocrine and neurosecretory systems of the adult mouse hypothalamic–pituitary axis. Adult male C57BL/6 J mice were analyzed using Western blotting, single-label immunoperoxidase histochemistry, and double-label immunofluorescence immunohistochemistry combined with hormone-specific markers. HAP1 immunoreactivity was detected throughout the pituitary gland, showing moderate scattered labeling in the anterior lobe, diffuse expression in the intermediate lobe, and intense accumulation in the posterior lobe. In the anterior and intermediate pituitary, HAP1 was highly expressed in thyrotrophs, corticotrophs, and melanotrophs, whereas gonadotrophs exhibited relatively low expression. In contrast, somatotrophs and lactotrophs lacked detectable HAP1 immunoreactivity. In the posterior pituitary, HAP1 was absent from S100β-positive pituicytes but strongly co-localized with oxytocin (OXT)- and arginine vasopressin (AVP)-containing neurosecretory structures, putative herring bodies. In the hypothalamus, HAP1 was prominently expressed in magnocellular neurons of the paraventricular and supraoptic nuclei and extensively co-localized with OXT- and AVP-producing neurons. Collectively, these findings demonstrate a highly cell-type-specific and compartmentalized distribution of HAP1 across the hypothalamic–pituitary axis. The continuous localization of HAP1 from hypothalamic neuronal somata to neurosecretory terminals provides an anatomical basis for investigating potential roles in vesicular trafficking, neuropeptide transport, and neurosecretory regulation, as well as its possible contribution to selective cellular resilience within neuroendocrine systems.

## 1 Introduction

Huntingtin-associated protein 1 (HAP1) is a cytoplasmic protein initially discovered for its interaction with huntingtin, the mutated gene product responsible for Huntington’s disease (Li et al., 1995). Two different HAP1 isoforms, HAP1A and HAP1B, are present in the rodent brain, each with a distinct C-terminal sequence. HAP1A forms characteristic dot-like structures in the cytoplasm called stigmoid bodies (STBs), while HAP1B shows a more diffuse distribution throughout the cytoplasm (Li et al., 1998; Gutekunst et al., 1998; Fujinaga et al., 2004, 2007; Islam et al., 2012, 2017, 2022; Yanai et al., 2020; Chen et al., 2020). STBs were initially described in our earlier studies as ovoid, non-membranous inclusions in the cytoplasm with moderate electron density (Shinoda et al., 1992, 1993). Previous research suggests that HAP1-positive STBs act as dynamic sites for sequestering intracellular proteins and may play roles in protein aggregation, trafficking, and maintaining neuronal homeostasis (Takeshita et al., 2006; Chen et al., 2020). In Huntington’s and several other neurodegenerative disorders (NDs), including spinal and bulbar muscular atrophy, spinocerebellar ataxia type 3, and Joubert syndrome, it has been established that STB/HAP1 functions as a protective barrier against apoptosis and cell death by increasing the threshold of vulnerability to neurodegeneration and giving neurons greater stability (Takeshita et al., 2006, 2011; Metzger et al., 2008).

Low or absent HAP1 expression in brain and spinal cord regions such as the striatum, thalamus, cerebral neocortex, cerebellum, and motor nuclei may contribute to their selective vulnerability to neurodegeneration in various NDs. However, NDs typically do not cause cell death in HAP1-enriched brain or spinal cord regions, such as the preoptic area, medial amygdala, hypothalamus, and autonomic nuclei. These findings gave rise to the “HAP1 protection hypothesis” against neurodegeneration (Kamei et al., 2001; Fujinaga et al., 2004, 2007, 2009, 2011; Islam et al., 2017, 2020a, 2022, 2025; Wroblewski et al., 2018; Wu et al., 2024; Afrin et al., 2025; Meher et al., 2025a). Beyond the brain and spinal cord, HAP1 expression has been reported in several neuroendocrine systems, including pancreatic islets, thyroid parafollicular cells, and adrenal chromaffin cells (Gutekunst et al., 1999; Liao et al., 2005, 2010; Mackenzie et al., 2014), suggesting a possible role in hormone secretion. Our previous study of rat pyloric glands revealed that HAP1 is specifically expressed in gastrin cells (Yanai et al., 2020), which are neuroendocrine cells responsible for gastrin synthesis and secretion.

The pituitary gland functions as the central endocrine organ within the hypothalamic–pituitary axis, integrating neural inputs from the hypothalamus to coordinate hormonal responses essential for growth, metabolism, reproduction, and stress adaptation (Melmed, 2020; Sheng et al., 2021). Hypothalamic nuclei, especially magnocellular and parvocellular systems, regulate pituitary hormone production and secretion through both vascular and neuronal routes via tightly controlled neuroendocrine signaling (Burbach et al., 2001; Iremonger and Power, 2025). Structurally, the pituitary gland is composed of the anterior (adenohypophysis), intermediate, and posterior (neurohypophysis) lobes, each containing distinct hormone-producing cell populations with specialized regulatory mechanisms and secretion profiles. The anterior pituitary consists of multiple endocrine cell types, including somatotrophs, lactotrophs, corticotrophs, thyrotrophs, and gonadotrophs, which secrete growth hormone (GH), prolactin (PRL), adrenocorticotropic hormone (ACTH), thyroid-stimulating hormone (TSH), and gonadotropins, including luteinizing hormone (LH) and follicle-stimulating hormone (FSH), respectively (Melmed, 2020). The intermediate lobe contains melanotrophs that produce melanocyte-stimulating hormone (MSH), a peptide derived from proopiomelanocortin (Cone, 2005; Millington, 2007). In contrast, the posterior pituitary lacks neurosecretory cell bodies; instead, it consists of specialized glial cells (pituicytes) and axon terminals from oxytocin (OXT)- and arginine vasopressin (AVP)-producing neurons originating in the hypothalamus. AVP and OXT are synthesized in magnocellular neurosecretory neurons of the supraoptic nucleus (SON) and paraventricular nucleus (PVN) and are transported along axons to the posterior lobe of the pituitary, where they are released into the systemic circulation. Notably, AVP and OXT immunoreactivity is not confined to neuronal cell bodies in the SON and PVN but is also prominently observed in axon terminals within the posterior pituitary, particularly in enlarged neurosecretory structures. These enlarged neurosecretory swellings, known as Herring bodies, serve as sites of hormone storage and release (Callewaere et al., 2006; Dayanithi et al., 2018).

HAP1 is highly enriched in several hypothalamic nuclei, where it has been implicated in the regulation of neuroendocrine functions (Gutekunst et al., 1998; Fujinaga et al., 2004). Functional studies have demonstrated that HAP1 plays a critical role in hypothalamic control of feeding behavior and energy homeostasis, as HAP1 deficiency leads to severe metabolic disturbances and postnatal lethality in mice (Li et al., 2003; Sheng et al., 2006, 2008). In addition, HAP1 has been shown to regulate receptor trafficking and neurotrophic signaling pathways, including modulation of TrkA receptor dynamics, thereby contributing to the maintenance of neuronal activity and neuroendocrine balance (Rong et al., 2007). Furthermore, HAP1 is involved in vesicular transport, cytoskeletal organization, and neuronal survival processes that are particularly important in highly active neurosecretory and endocrine cells (Engelender et al., 1997; Li and Li, 2004; Yanai et al., 2020). Despite its established presence in hypothalamic regions that regulate pituitary function, the expression and cellular localization of HAP1 within the pituitary gland itself remain poorly characterized. It remains unknown whether HAP1 exhibits cell-type-specific localization across distinct endocrine and neurosecretory compartments of the hypothalamic–pituitary axis. Previous work by Liao et al. (2005) briefly reported HAP1 immunoreactivity in the rat pituitary gland. However, whether HAP1 is differentially expressed among hormone-producing cell types across the anterior, intermediate, and posterior lobes, or contributes to hormone storage and secretion in endocrine and neurosecretory compartments, remains unclear.

In this study, we investigated the detailed expression patterns and immunohistochemical phenotypes of HAP1 across the adult mouse hypothalamic–pituitary axis. Using Western blotting and immunohistochemistry combined with hormone-specific markers, we examined the localization of HAP1 in distinct endocrine and neurosecretory cell populations of the anterior, intermediate, and posterior pituitary, as well as in magnocellular hypothalamic nuclei, to determine whether HAP1 exhibits a continuous distribution along the hypothalamic–neurohypophyseal axis. Defining the spatial distribution of HAP1 within this neuroendocrine system may provide new insights into its roles in vesicular trafficking and neuropeptide transport, while offering an anatomical framework for understanding selective cellular vulnerability within the hypothalamic–pituitary axis.

## 2 Materials and methods

### 2.1 Animals

Ten-week-old adult male C57BL/6 J mice were obtained from Japan SLC Inc. (Shizuoka, Japan). Male mice were used to establish the baseline cell-type-specific distribution of HAP1 while avoiding potential variability associated with estrous-cycle-dependent hormonal fluctuations in females. The present study was not designed to assess sex differences; therefore, the findings are restricted to adult male mice. Animals were housed in standard laboratory cages under controlled environmental conditions, with ambient temperature maintained at 22–24 °C. Mice were provided with a standard pellet diet and had free access to water ad libitum. A 12 h light/dark cycle was maintained (lights on from 08:00 to 20:00). A total of 30 mice were used in this study, and all efforts were made to minimize animal suffering and to reduce the number of animals required. Of these, 12 mice were used for Western blotting, 6 mice for single-label immunoperoxidase analysis of HAP1 distribution, and 12 mice for double-label immunofluorescence characterization and quantitative co-localization analysis. For Western blotting, three independent experiments were performed using separate tissue preparations; in each experiment, pituitary glands from four mice were pooled for protein extraction, whereas hypothalamic tissue from one of the same four mice was processed individually. For double-label immunofluorescence, six mice from the 12-animal cohort were analyzed for each HAP1/marker combination (*n* = 6 biological replicates). The subsets of six mice could overlap among different HAP1/marker combinations because of the limited number of usable sections obtainable from each mouse pituitary. For all procedures, mice were deeply anesthetized using a combination of medetomidine, midazolam, and butorphanol (MMB). The anesthetic agents were administered at doses of 0.3 mg/kg medetomidine (Domitor^®^, Orion Pharma, Espoo, Finland), 4.0 mg/kg midazolam (Midazolam^®^, Sandoz, Basel, Switzerland), and 5.0 mg/kg butorphanol (Vetorphale^®^, Meiji Seika Pharma, Tokyo, Japan), as previously described (Jahan et al., 2025; Meher et al., 2025b). The mixture was delivered via intraperitoneal injection at a volume of 0.20–0.25 mL per animal. Adequate depth of anesthesia was confirmed by the absence of a withdrawal reflex in response to a tail pinch.

### 2.2 Antibodies

Details of the antibodies used in this study are summarized in Table 1 (primary antibodies) and Table 2 (secondary antibodies). All antibodies were commercially obtained. Their specificity and characterization have been previously validated either in our earlier studies or in reports by other investigators. In addition, the specificity of the anti-HAP1 antibody was further confirmed in the present study using a pre-adsorption control assay (Figure 1).

**Table 1:** Primary antibodies used in this study.

Antibody	Immunogen	Code	Host/Clonality	Source	Dilution	References
HAP1	Rat HAP1 C-terminus	Cat# sc-8770, RRID: AB_647322	Goat polyclonal	Santa Cruz Biotechnology, Santa Cruz, CA	1:10,000	Islam et al. (2012, 2025)
ACTH	Synthetic ACTH (1–24) bound to KLH with carbodiimide	Cat# AB902, RRID: AB_2166019	Rabbit polyclonal	Merck Millipore, Temecula, CA, USA	1:5,000	Sakai et al. (2003)
TSH	Purified bovine thyroid-stimulating hormone	Cat# AB976, RRID: AB_11210767	Rabbit polyclonal	Merck Millipore, Temecula, CA, USA	1:10,000	Nishimura et al. (2003)
MSH	Synthetic α-MSH conjugated to bovine thyroglobulin using glutaraldehyde	Cat# 20074, RRID: AB_572218	Rabbit polyclonal	Immunostar, Hudson, WI, USA	1:2,000	Kawabe et al. (2012)
LH	N-epitope synthetic peptide taken within amino acid region 1–50 on human LH protein	Cat# LH-101AP, RRID: AB_3741504	Rabbit polyclonal	FabGennix International, Inc., Frisco, TX, USA	1:2,000	Park et al. (2017)
FSH	FSH beta	Cat# ab281562, RRID: AB_2928119	Rabbit monoclonal	Abcam, Cambridge, UK	1:1,000	Huang et al. (2024)
GH	Recombinant Full-length protein corresponding to Rat Gh1	Cat#ab126882, RRID: AB_11128284	Rabbit polyclonal	Abcam, Cambridge, UK	1:2,000	Lombardero et al. (2020)
PRL	Synthetic peptide within mouse PRL amino acids 150 to the C-terminus	Cat#ab183967, RRID: AB_2814839	Rabbit monoclonal	Abcam, Cambridge, UK	1:2,000	Hidema et al. (2024)
OXT	Synthetic oxytocin conjugated to thyroglobulin	Cat# AB911, RRID: AB_2313719	Rabbit polyclonal	Merck Millipore, Temecula, CA, USA	1:1,000	Nishimura et al. (2020)
AVP	Native full-length protein corresponding to rat Avp	Cat# ab39363, RRID: AB_778778	Rabbit polyclonal	Abcam, Cambridge, UK	1:1,000	Zhao et al. (2017)
S100β	Recombinant protein corresponding to amino acids 1–92 of rat S100β	Cat# 287006, RRID: AB_2713986	Rabbit polyclonal	Synaptic Systems, Göttingen, Germany	1:1,000	Lee et al. (2023)
Anti-α-tubulin	Microtubule derived from chicken embryonic brain	Cat# T6199, RRID: AB_477583	Mouse monoclonal	Sigma-Aldrich, St. Louis, MO, USA	1:200,000	Islam et al. (2021)

ACTH, Adrenocorticotropic Hormone; HAP1, huntingtin-associated protein 1; KLH, keyhole limpet hemocyanin; GH, growth hormone; PRL, prolactin; TSH, thyroid-stimulating hormone; LH, luteinizing hormone; FSH, follicle-stimulating hormone; MSH, melanocyte-stimulating hormone; AVP, arginine vasopressin, OXT, oxytocin; RRID, research resource identifier.

**Table 2:** Secondary antibodies used in this study.

Secondary antibody and other reagents	Immunogen	Host	Catalogue number/Research resource identifiers (RRIDs)/Source	Application	Dilution	References
HRP-labelled anti-goat	Goat IgG, F(ab’) 2	Donkey	Cat# 3851, RRID: AB_641200 Santa Cruz Biotechnology, Santa Cruz, CA, USA.	Western blotting	1:10,000	Islam et al. (2025)
HRP-labelled anti-mouse	Mouse IgG whole molecule	Sheep	Cat# NA931, RRID: AB_772210 GE Healthcare, Buckinghamshire, UK.		1:20,000	Islam et al. (2022)
Biotinylated donkey anti-goat	IgG, isolated from goat serum	Donkey	Cat# AP180B, RRID: AB_92569 Millipore, Darmstadt, Germany.	Immunoperoxidase staining	1:1,000	Islam et al. (2017)
Alexa Fluor 594 donkey anti-goat	Goat IgG (H + L)	Donkey	Cat# A-11058, RRID: AB_2534105 Invitrogen, Eugene, OR, USA.	Immunofluorescence staining	1:1,000	Tarif et al. (2021, 2023)
Alexa Fluor 488 donkey anti-rabbit	Rabbit IgG (H + L)	Donkey	Cat# A32790, RRID: AB_2762833 Invitrogen, Eugene, OR, USA.		1:1,000	Jahan et al. (2025)
HRP-conjugated streptavidin	N/A	N/A	Cat# P0397, Agilent Dako, Glostrup, Denmark.	Immunoperoxidase staining	1:1,000	Yanai et al. (2020)

HRP, horseradish peroxidase; IgG, immunoglobulin G; RRID, research resource identifier.

**Figure 1:**
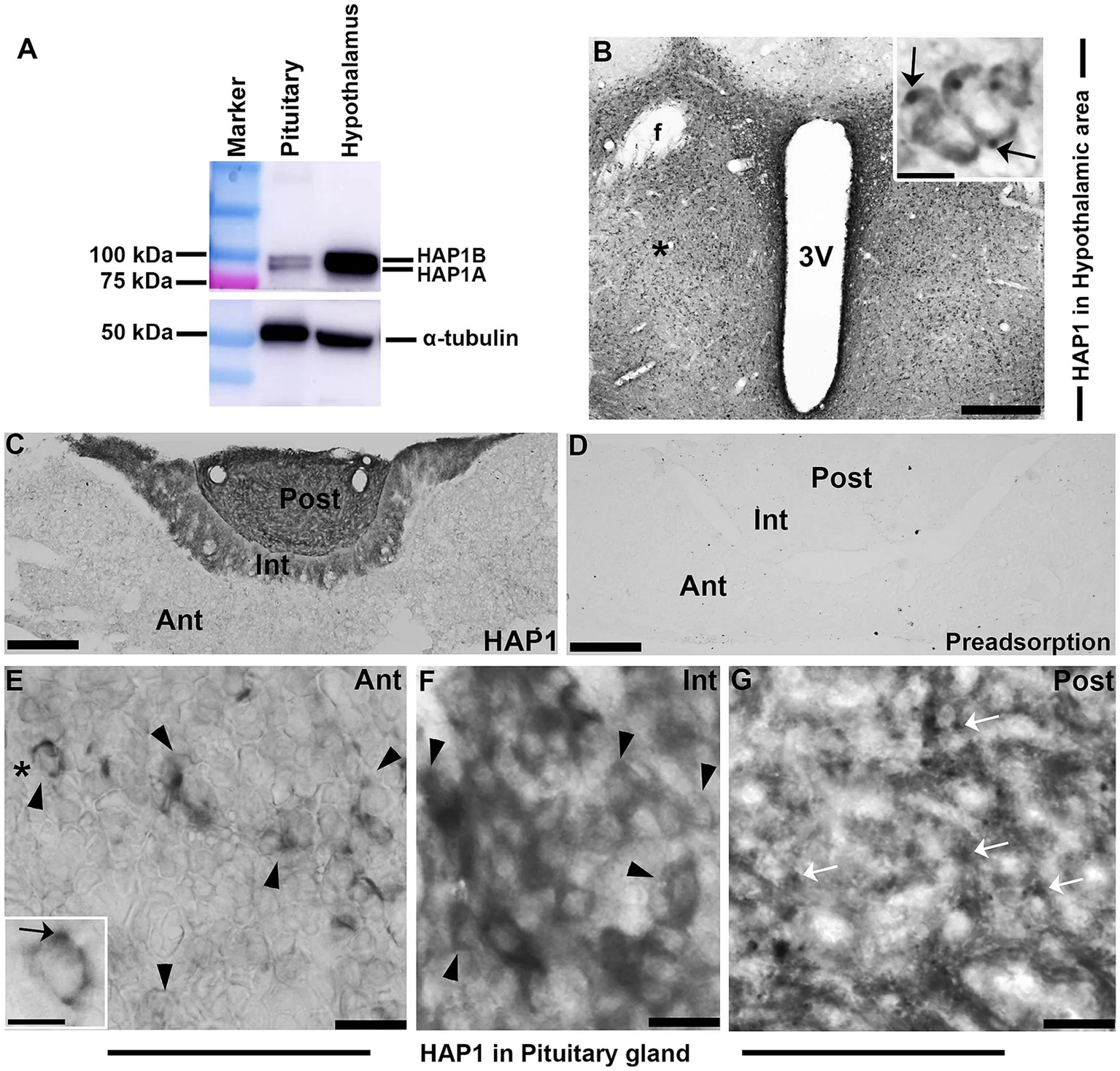
General expression of HAP1 in the mouse pituitary gland. **(A)** Western blot analysis showing the two HAP1 isoforms (HAP1A and HAP1B) in the pituitary gland and hypothalamus (positive control). α-tubulin was used as a loading control. **(B)** Immunohistochemical localization of HAP1 in the hypothalamic region surrounding the third ventricle (3 V). The inset shows a higher magnification of the area indicated by the asterisk (*); Black arrows indicate stigmoid bodies (STBs). **(C)** Low-magnification view of the pituitary gland showing HAP1 immunoreactivity in the anterior (Ant), intermediate (Int), and posterior (Post) lobes. **(D)** Preadsorption control demonstrating the absence of HAP1 immunoreactivity. **(E–G)** Higher-magnification images of the anterior **(E)**, intermediate **(F)**, and posterior **(G)** pituitary lobes. Arrowheads indicate cytoplasmic HAP1 immunoreactivity in scattered cells. Inset: Black arrow indicates STB. White arrows indicate enlarged HAP1-immunoreactive neurosecretory structures. f, fornix. Scale bars: 400 μm in **B**; 200 μm in **C,D**; 25 μm in **E–G**; and 10 μm in the insets.

### 2.3 Western blotting

Western blot analysis was performed as described in our previous study (Oka et al., 2017). Briefly, pituitary gland and hypothalamic tissues were collected. Western blotting was independently performed three times using separate tissue preparations. For each experiment, pituitary glands from four mice were pooled to obtain sufficient tissue for protein extraction, whereas hypothalamic tissue from one mouse within the corresponding group was processed individually. The pooled pituitary glands and individual hypothalamic tissues were homogenized separately in RIPA lysis buffer supplemented with a protease inhibitor cocktail (sc-24948; Santa Cruz Biotechnology, Dallas, TX, USA). The homogenates were centrifuged at 13,000 × g for 10 min at 4 °C, and the supernatants were collected as protein extracts. Protein concentrations were determined using the Bradford Protein Assay Kit (Bio-Rad Laboratories, Hercules, CA, USA). Protein samples were mixed with 1 M dithiothreitol and heated at 95 °C for 5 min prior to electrophoresis. Protein samples (20 μg per lane) were separated by SDS–PAGE on 10% polyacrylamide gels and subsequently transferred onto polyvinylidene difluoride membranes using a wet transfer system for 2 h. Following electrophoretic transfer, the same membrane was horizontally divided according to molecular-weight range. The upper membrane portion was processed for HAP1 immunodetection, whereas the lower portion was processed separately for α-tubulin immunodetection. The membrane portions were blocked for 1 h at room temperature in 5% non-fat milk prepared in phosphate-buffered saline containing Tween 20 (PBST), followed by overnight incubation at 4 °C with goat polyclonal anti-HAP1 (1:10,000) or mouse monoclonal anti-α-tubulin (1:200,000) antibodies (Table 1). After washing with PBST, the membrane portions were incubated with the corresponding horseradish peroxidase-conjugated secondary antibodies (anti-goat, 1:10,000; anti-mouse, 1:20,000; Table 2) for 2 h at 20 °C. Immunoreactive bands were visualized using Amersham ECL Prime Detection Reagent (Cytiva, Marlborough, MA, USA) and detected with an Amersham Imager 600 system (GE Healthcare). Uncropped original membrane images, including molecular-weight marker lanes and additional protein-loading amounts (10 and 5 μg per lane), are provided in Supplementary Figure S1.

### 2.4 Tissue processing and sectioning for immunohistochemistry

Mice were deeply anesthetized with the MMB mixture as described above and transcardially perfused with ice-cold saline, followed by 4% paraformaldehyde in 0.1 M phosphate buffer (PB; pH 7.4). The brain and pituitary gland were carefully dissected and post-fixed in the same fixative for 24 h at 4 °C. Subsequently, tissues were cryoprotected by immersion in 30% sucrose prepared in 0.1 M PB until they sank. The brain and pituitary gland were then sectioned at 30 μm using a cryostat (Moriyasu-Konetsu, Osaka, Japan) equipped with a microtome (Microm, Walldorf, Germany). Sections were stored at 4 °C in 0.02 M phosphate-buffered saline (PBS; pH 7.4) containing 0.1% sodium azide until further processing for immunohistochemistry.

### 2.5 Single-label immunoperoxidase staining

Single-label immunoperoxidase staining was performed as described in our previous studies (Islam et al., 2020b, 2021, 2023). Briefly, free-floating sections of the pituitary gland and the brain (including hypothalamic regions) were incubated in blocking solution containing 10% normal donkey serum (NDS; Millipore, Temecula, CA, USA) with 0.3% Triton X-100 for 2 h at 4 °C. Sections were then treated with a solution of 1.5% hydrogen peroxide in 50% methanol at 4 °C for 30 min to quench endogenous peroxidase activity. Following this, sections were incubated with primary antibody against HAP1 (1:10,000; Table 1) at 20 °C for 3 days. For the pre-adsorption control, the diluted primary antibody was preincubated overnight at 4 °C with the corresponding blocking peptide. After thorough washing, sections were incubated with biotinylated donkey anti-goat secondary antibody (Millipore; 1:1,000; Table 2) for 2–3 h at 20 °C, followed by incubation with peroxidase-conjugated streptavidin (Dako, Glostrup, Denmark; 1:1,000; Table 2) for an additional 2 h at 20 °C. After washing in 0.05 M Tris–HCl buffer (pH 7.6), immunoreactivity was visualized using a nickel-enhanced diaminobenzidine (DAB) reaction. Sections were incubated at 4 °C for 10–15 min in a solution containing 0.02% 3,3′DAB (Dojindo Laboratories, Kumamoto, Japan), 0.6% nickel ammonium sulfate (Sigma–Aldrich, Tokyo, Japan), and 0.0008% hydrogen peroxide in Tris–HCl buffer. Finally, sections were mounted onto glass slides, air-dried, dehydrated through a graded ethanol series followed by xylene, and coverslipped using Entellan New (Millipore).

### 2.6 Double-label immunofluorescence staining

Double-label immunofluorescence staining was performed as described in our previous studies (Jahan et al., 2015; Islam et al., 2025). Briefly, free-floating sections of the pituitary gland and hypothalamic regions were incubated in 10% NDS with 0.3% Triton X-100 for 3 h at 4 °C. Sections were then incubated with a combination of primary antibodies consisting of goat anti-HAP1 (1:10,000) together with one of the following rabbit antibodies (Table 1 for details): anti-TSH (1:10,000), anti-ACTH (1:5,000), anti-MSH (1:2,000), anti-GH (1:2,000), anti-PRL (1:2,000), anti-LH (1:2,000), anti-FSH (1:1,000), anti-AVP (1:1,000), anti-OXT (1:1,000), or anti-S100β (1:1,000). Primary antibody incubation was carried out at 20 °C for 3 days. After washing with PBS, sections were incubated with a mixture of secondary antibodies: Alexa Fluor 594-conjugated donkey anti-goat IgG (Invitrogen, Eugene, OR, USA; 1:1,000) and Alexa Fluor 488-conjugated donkey anti-rabbit IgG (Invitrogen, Rockford, IL, USA; 1:1,000) for 3 h at 20 °C. Nuclear counterstaining was performed using DAPI, particularly for sections of the posterior pituitary. Following additional washes, sections were mounted onto glass slides, air-dried for approximately 60 min, and coverslipped with Fluoromount/Plus (Diagnostic Biosystems, Pleasanton, CA, USA).

### 2.7 Photomicrograph acquisition

Images of immunoperoxidase-stained sections were captured using an STC-MCS500U3V USB 3.0 color digital camera (Omron Sentech Co., Ltd., Kanagawa, Japan) mounted on a Nikon Eclipse E80i microscope (Nikon Corporation, Tokyo, Japan). Immunofluorescence images were acquired using a Leica Stellaris 8 CRS inverted confocal laser scanning microscope (Leica Microsystems, Wetzlar, Germany) equipped with an HC PL APO CS2 40×/1.30 NA oil-immersion objective. Alexa Fluor 488 and Alexa Fluor 594 fluorescence signals were acquired sequentially using 499- and 590-nm excitation, respectively, with corresponding detection ranges of 507–595 nm and 595–750 nm. Images were acquired at 1024 × 1,024 pixels with a pixel size of 0.284 μm, a pinhole setting of 1 Airy unit (59.7 μm), a scan speed of 100 Hz, and a pixel dwell time of 7.69 μs. Line and frame averaging were each set to 1. Image acquisition parameters were kept constant across samples within each HAP1/marker comparison to ensure valid comparisons.

### 2.8 Tissue analysis and cell quantification

For tissue analysis, immunofluorescence images were imported into ImageJ software (NIH, Bethesda, MD, USA) for quantitative evaluation. For cellular and neuronal analyses, only cells with clearly identifiable nuclei were included in the analysis. Immunoreactive cells were defined as positive when fluorescence intensity exceeded 1.5–2 standard deviations above the mean background fluorescence of unlabeled cells (Kestell et al., 2015; Islam et al., 2020a). Co-localization analyses (HAP1/marker and marker/HAP1) were performed using four sections per pituitary or hypothalamic region from each animal. The sections, separated by at least 60 μm, were selected to minimize oversampling of the same cells or structures. For each HAP1/marker pair, the HAP1/marker ratio was defined as the percentage of marker-immunoreactive cells, neurons, or structures that were also HAP1-immunoreactive, whereas the marker/HAP1 ratio was defined as the percentage of HAP1-immunoreactive cells, neurons, or structures that were also immunoreactive for the corresponding marker. These reciprocal co-localization ratios were calculated based on the numbers of HAP1-immunoreactive elements, marker-immunoreactive elements, and double-labeled elements, following previously established methods (Nagano and Shinoda, 1994; Jahan et al., 2015). Because each HAP1/marker combination was examined in a separate double-label experiment, the HAP1-positive denominator was independently determined for each marker. Therefore, marker/HAP1 ratios obtained for different markers represent independently sampled HAP1-positive populations rather than simultaneous phenotyping of the same individual HAP1-positive elements. Although numerical sums of these ratios may exceed 100%, such sums alone cannot determine whether the values reflect biological overlap among marker-defined populations or variability introduced by independent sampling and staining experiments. A total of 6 mice were included in the quantitative analysis. Data are presented as mean ± SEM (*n* = 6).

## 3 Results

### 3.1 Expression and regional localization of HAP1 in the pituitary gland

Western blot analysis revealed the presence of HAP1 protein in the pituitary gland of adult mice (Figure 1A). Although HAP1 expression was substantially higher in the hypothalamus (control) than in the pituitary gland, both HAP1A and HAP1B isoforms were clearly detected in the latter (Figure 1A). Immunohistochemical examination showed strong diffuse cytoplasmic HAP1 immunoreactivity in the hypothalamus (Figure 1B), with distinct punctate STBs. In the pituitary gland, HAP1 immunoreactivity displayed a lobe-specific distribution pattern (Figure 1C). No staining was observed in sections processed with the preadsorbed antibody, confirming the specificity of the HAP1 antibody (Figure 1D). The anterior lobe exhibited moderate, scattered cytoplasmic HAP1 immunoreactivity (Figure 1E). At higher magnification, STBs were detected within the cytoplasm of HAP1-immunoreactive anterior pituitary cells (Figure 1E). Together with the Western blot results, these findings indicate that both HAP1A and HAP1B isoforms are expressed in the pituitary gland. The intermediate lobe exhibited stronger diffuse cytoplasmic HAP1 immunoreactivity than the anterior lobe, making individual STBs difficult to distinguish by light microscopy. Although punctate HAP1-positive structures were occasionally observed, they were largely obscured by the intense diffuse cytoplasmic immunoreactivity (Figure 1F). The posterior lobe exhibited intense HAP1 immunoreactivity characterized by dense labeling of enlarged punctate neurosecretory structures (Figure 1G).

### 3.2 High expression of HAP1 in thyrotrophs, corticotrophs, and melanotrophs

Double immunofluorescence analysis revealed extensive co-localization of HAP1 with TSH-immunoreactive cells (Figures 2A–C). Quantitative analysis showed that 82.36% of TSH-positive cells were HAP1-positive, whereas 76.21% of HAP1-immunoreactive cells exhibited TSH immunoreactivity (Figure 2D, Table 3), indicating strong co-localization of HAP1 with thyrotrophs. Similarly, ACTH-immunoreactive cells exhibited strong co-localization with HAP1 (Figures 2E–G), with 67.49% of ACTH-positive cells expressing HAP1 and 81.25% of HAP1-immunoreactive cells co-expressing ACTH (Figure 2H, Table 3). In the intermediate lobe, MSH-producing cells exhibited the highest degree of co-localization with HAP1 (Figures 2I–K), with 87.41% of MSH-positive cells expressing HAP1 and 91.72% of HAP1-immunoreactive cells exhibiting MSH immunoreactivity (Figure 2L, Table 3). Collectively, these findings demonstrate that HAP1 is highly enriched in thyrotrophs, corticotrophs, and melanotrophs of the anterior and intermediate pituitary.

**Figure 2:**
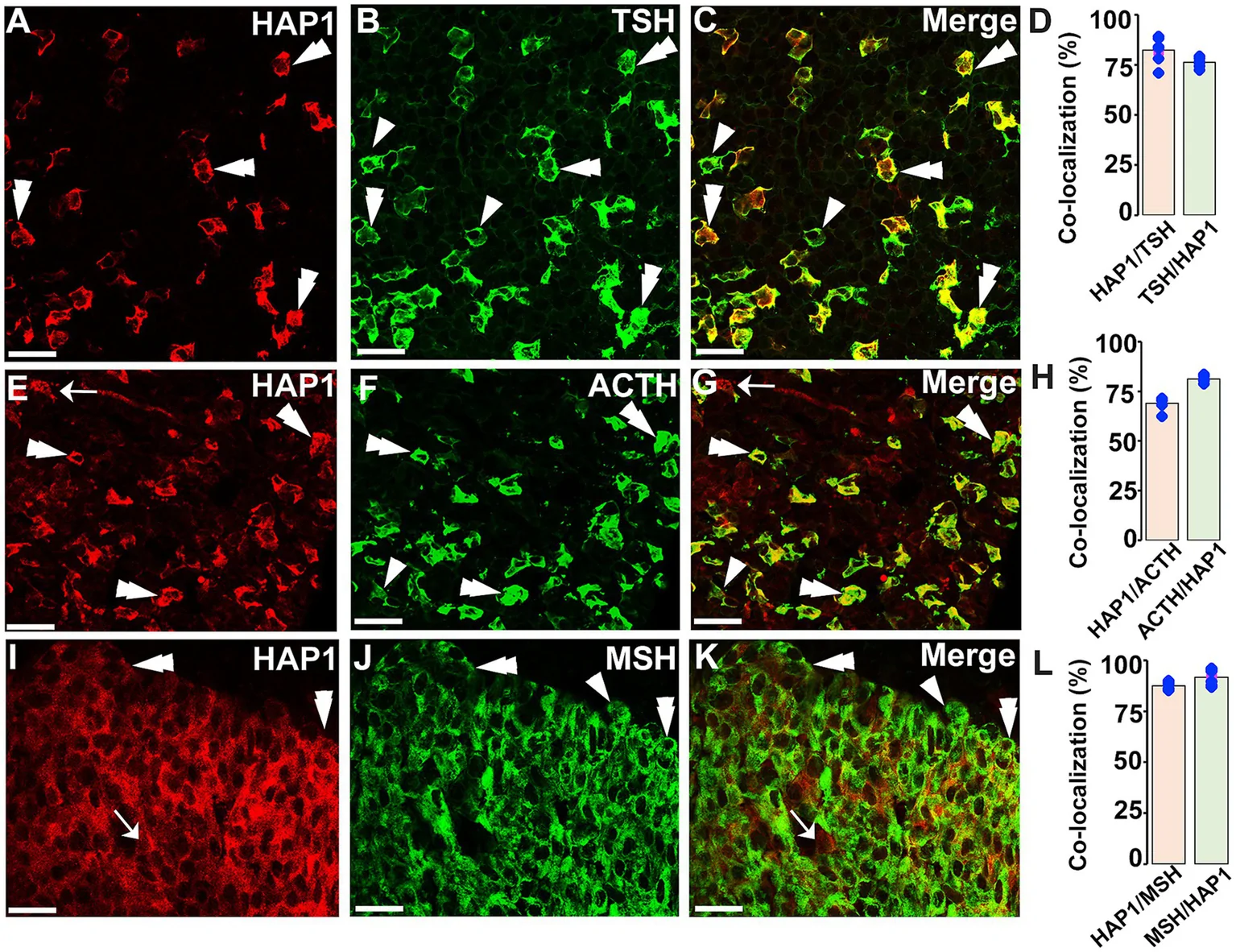
Double immunofluorescence staining of HAP1 with TSH-, ACTH-, and MSH-immunoreactive cells in the mouse pituitary gland. **(A–C)** Anterior pituitary sections stained for HAP1 (red; **A**) and TSH (green; **B**). **(C)** Merged image showing co-localization of HAP1 and TSH. **(D)** Quantitative analysis showing the reciprocal co-localization ratios between HAP1 and TSH (HAP1/TSH and TSH/HAP1). **(E–G)** Anterior pituitary sections stained for HAP1 (red; **E**) and ACTH (green; **F**). **(G)** Merged image showing co-localization of HAP1 and ACTH. **(H)** Quantitative analysis showing the reciprocal co-localization ratios between HAP1 and ACTH (HAP1/ACTH and ACTH/ACTH). **(I–K)** Intermediate pituitary sections stained for HAP1 (red; **I**) and MSH (green; **J**). **(K)** Merged image showing co-localization of HAP1 and MSH. **(L)** Quantitative analysis showing the reciprocal co-localization ratios between HAP1 and MSH (HAP1/MSH and MSH/HAP1). In **D**, **H**, and **L**, individual data points represent biological replicates (*n* = 6 mice), and bars indicate mean ± *SEM*. For each HAP1/marker pair, HAP1/marker represents the percentage of marker-positive cells that were HAP1-positive, whereas marker/HAP1 represents the percentage of HAP1-positive cells that were marker-positive within the corresponding double-label experiment. Reciprocal co-localization ratios were calculated independently for each HAP1/marker double-label experiment and therefore do not represent mutually exclusive fractions of a common HAP1-positive cell population. Arrows indicate single-labeled HAP1-positive cells, arrowheads indicate single-labeled TSH-, ACTH-, or MSH-positive cells, and double arrowheads indicate cells co-labeled for HAP1 and the corresponding marker. Scale bar = 20 μm. HAP1, huntingtin-associated protein 1; TSH, thyroid-stimulating hormone; ACTH, adrenocorticotropic hormone; MSH, melanocyte-stimulating hormone.

**Table 3:** Co-localization ratios of HAP1 and cell-specific markers across the hypothalamic–pituitary axis.

Hypothalamic–pituitary axis		Cell-specific markers	HAP1/marker co-localization ratio (%)	Marker/HAP1 co-localization ratio (%)
Pituitary gland	Anterior lobe	GH	0.00	0.00
		PRL	0.00	0.00
		LH	29.33 ± 3.85	57.94 ± 4.17
		FSH	10.34 ± 2.43	18.75 ± 2.81
		TSH	82.36 ± 2.77	76.21 ± 1.03
		ACTH	67.49 ± 1.75	81.25 ± 0.69
	Intermediate lobe	MSH	87.41 ± 0.74	91.72 ± 1.67
	Posterior lobe	OXT	76.68 ± 3.29	93.42 ± 3.18
		AVP	71.53 ± 2.74	92.37 ± 3.66
		S100β	0.00	0.00
Hypothalamus	Paraventricular nucleus	OXT	95.23 ± 1.21	69.53 ± 1.18
		AVP	95.11 ± 2.29	63.78 ± 1.12
	Supraoptic nucleus	OXT	97.29 ± 0.87	92.31 ± 0.91
		AVP	94.44 ± 1.76	73.39 ± 1.02

Values are presented as mean ± SEM (%) (*n* = 6 mice). For each HAP1/marker pair, HAP1/marker represents the percentage of marker-positive cells, neurons, or structures that were HAP1-positive, whereas marker/HAP1 represents the percentage of HAP1-positive cells, neurons, or structures that were positive for the corresponding marker. Each HAP1/marker combination was examined in a separate double-label experiment, and the HAP1-positive denominator was therefore independently determined for each marker. Thus, marker/HAP1 ratios for different markers represent independently sampled HAP1-positive populations rather than simultaneous phenotyping of the same individual HAP1-positive elements. Numerical sums exceeding 100% may be compatible with biological overlap among marker-defined populations, but such sums alone cannot establish biological overlap among the same individual HAP1-positive elements.

### 3.3 Limited expression of HAP1 in gonadotrophs

Double immunofluorescence analysis further demonstrated that HAP1 expression was relatively low in gonadotrophs of the anterior pituitary. LH-immunoreactive cells exhibited only limited co-localization with HAP1 (Figures 3A–C). Quantitative analysis showed that 29.33% of LH-positive cells expressed HAP1, whereas 57.94% of HAP1-immunoreactive cells exhibited LH immunoreactivity (Figure 3D, Table 3). Similarly, FSH-immunoreactive cells displayed minimal overlap with HAP1 (Figures 3E–G), with only 10.34% of FSH-positive cells expressing HAP1 and 18.75% of HAP1-immunoreactive cells co-expressing FSH (Figure 3H, Table 3). Collectively, these findings indicate that HAP1 expression is comparatively sparse in gonadotrophs, particularly in FSH-producing cells.

**Figure 3:**
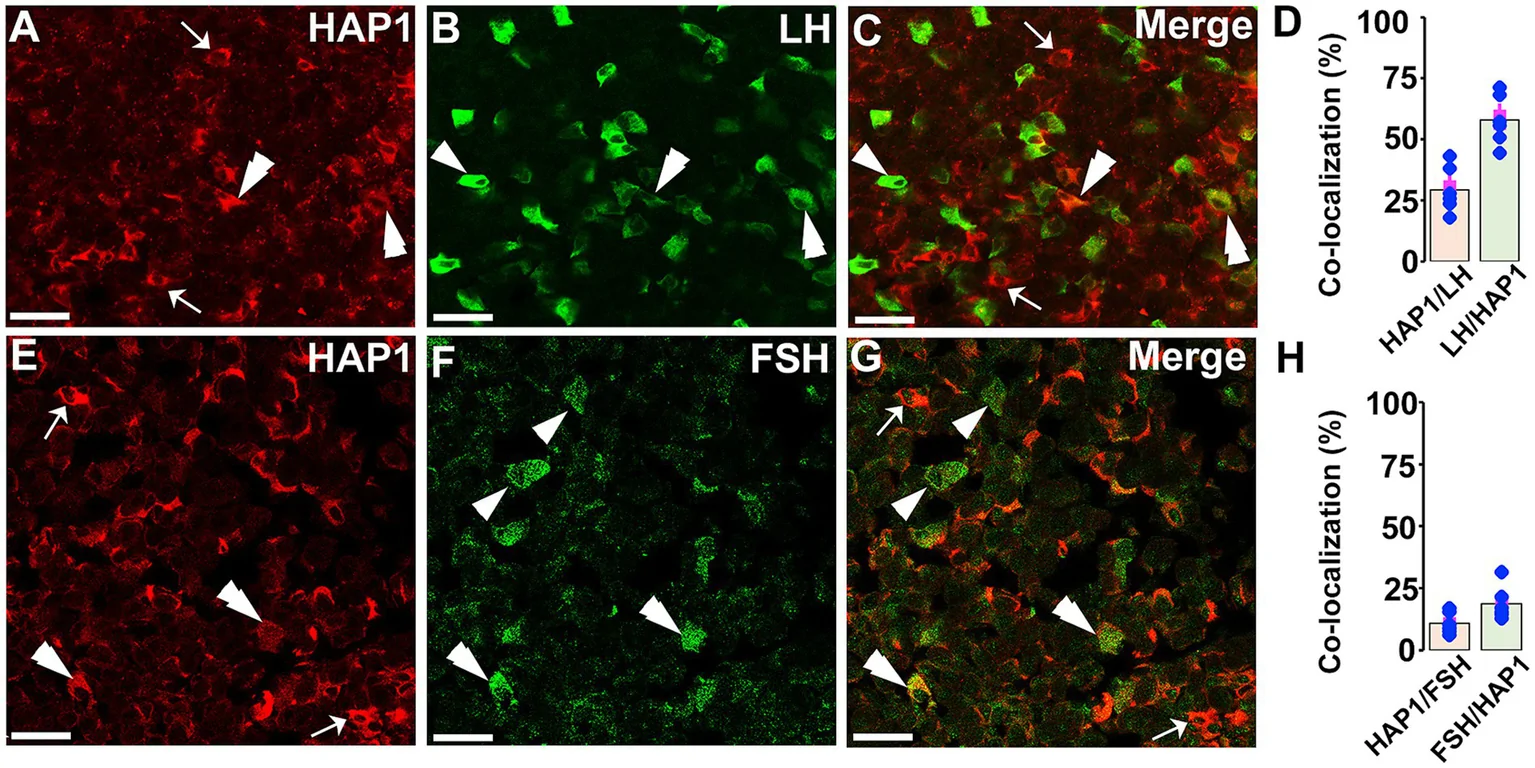
Double immunofluorescence staining of HAP1 with LH- and FSH-immunoreactive cells in the mouse anterior pituitary. **(A–C)** Anterior pituitary sections stained for HAP1 (red; **A**) and LH (green; **B**). **(C)** Merged image showing co-localization of HAP1 and LH. **(D)** Quantitative analysis showing the reciprocal co-localization ratios between HAP1 and LH (HAP1/LH and LH/HAP1). **(E–G)** Anterior pituitary sections stained for HAP1 (red; **E**) and FSH (green; **F**). **(G)** Merged image showing co-localization of HAP1 and FSH. **(H)** Quantitative analysis showing the reciprocal co-localization ratios between HAP1 and FSH (HAP1/FSH and FSH/FSH1). In **D,H**, individual data points represent biological replicates (*n* = 6 mice), and bars indicate mean ± SEM. For each HAP1/marker pair, HAP1/marker represents the percentage of marker-positive cells that were HAP1-positive, whereas marker/HAP1 represents the percentage of HAP1-positive cells that were marker-positive within the corresponding double-label experiment. Reciprocal co-localization ratios were calculated independently for each HAP1/marker double-label experiment and therefore do not represent mutually exclusive fractions of a common HAP1-positive cell population. Arrows indicate single-labeled HAP1-positive cells, arrowheads indicate single-labeled LH- or FSH-positive cells, and double arrowheads indicate cells co-labeled for HAP1 and the corresponding marker. Scale bar = 20 μm. HAP1, huntingtin-associated protein 1; LH, luteinizing hormone; FSH, follicle-stimulating hormone.

### 3.4 Absence of HAP1 in somatotrophs and lactotrophs

In contrast to other anterior pituitary endocrine cell types, double immunofluorescence analysis revealed no detectable HAP1 immunoreactivity in GH- or PRL-producing cells (Figures 4A–C, E–G)). HAP1-, GH-, and PRL-immunoreactive cells formed distinct, non-overlapping cell populations, with no merged signals observed (Figures 4C,G). Quantitative analysis confirmed the absence of co-localization, with 0.0% overlap between HAP1- and GH-positive cells (Figure 4D, Table 3) and between HAP1- and PRL-positive cells (Figure 4H, Table 3).

**Figure 4:**
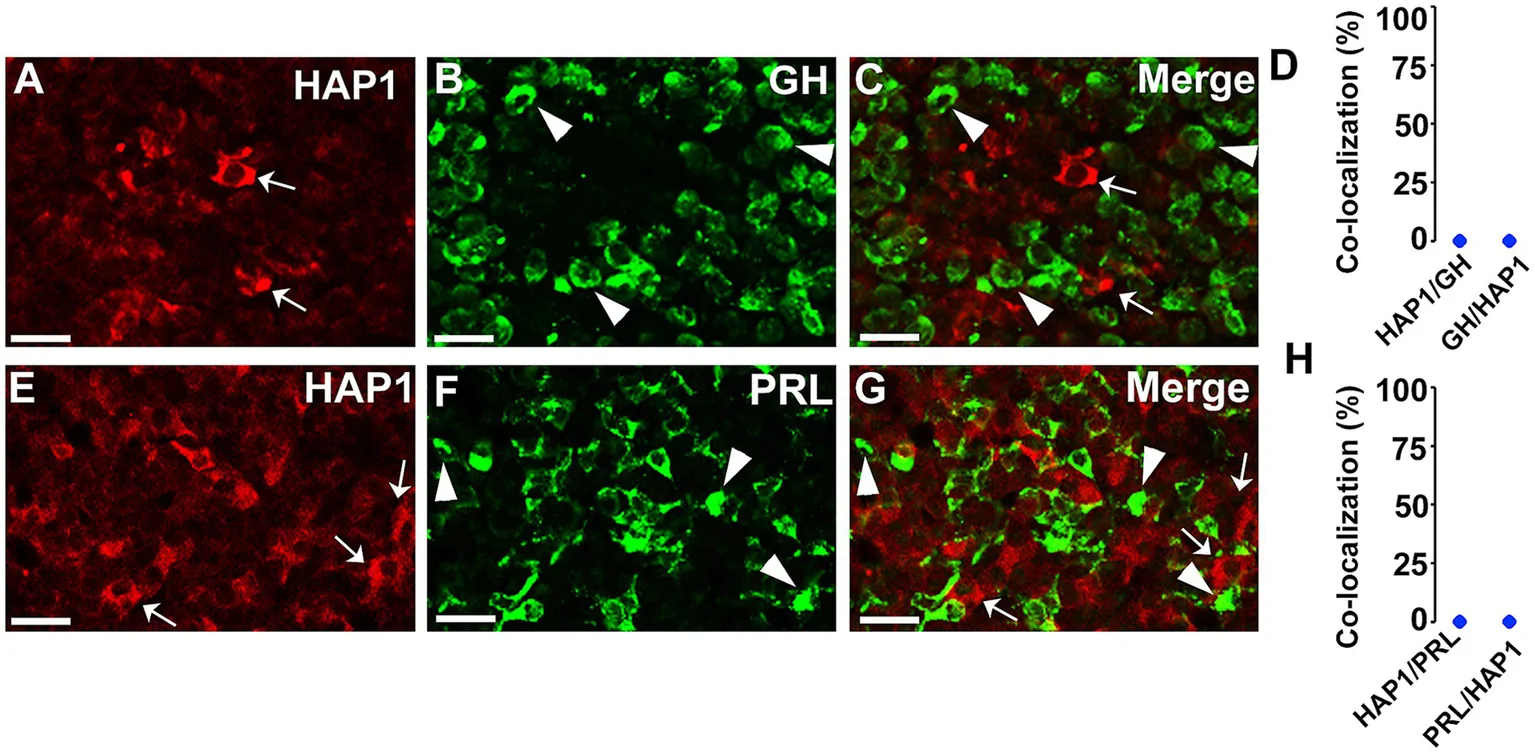
Double immunofluorescence staining of HAP1 with GH- and PRL-immunoreactive cells in the mouse anterior pituitary. **(A–C)** Anterior pituitary sections stained for HAP1 (red; **A)** and GH (green; **B)**. **(C)** Merged image showing no detectable co-localization of HAP1 and GH. **(D)** Quantitative analysis confirming the absence of reciprocal co-localization between HAP1 and GH (HAP1/GH and GH/HAP1; 0.0% for both ratios). **(E–G)** Anterior pituitary sections stained for HAP1 (red; **E**) and PRL (green; **F**). **(G)** Merged image showing no detectable co-localization of HAP1 and PRL. **(H)** Quantitative analysis confirming the absence of reciprocal co-localization between HAP1 and PRL (HAP1/PRL and PRL/HAP1; 0.0% for both ratios). In **D,H**, individual data points represent biological replicates (*n* = 6 mice). All six individual values were 0%; consequently, the individual data points are superimposed at 0%. HAP1/marker represents the percentage of marker-positive cells that were HAP1-positive, whereas marker/HAP1 represents the percentage of HAP1-positive cells that were marker-positive within the corresponding double-label experiment. Arrows indicate single-labeled HAP1-positive cells, arrowheads indicate single-labeled GH- or PRL-positive cells. Scale bar = 20 μm. HAP1, huntingtin-associated protein 1; GH, growth hormone; PRL, prolactin.

Compared with the high HAP1 co-localization observed in thyrotrophs, corticotrophs, and melanotrophs (Figure 2), gonadotrophs exhibited markedly lower levels of HAP1 co-localization (Figure 3). In contrast, GH- and PRL-producing cells showed no detectable co-localization with HAP1 (Figure 4). Together, these findings demonstrate a striking cell-type-specific distribution of HAP1 within the adenohypophysis and intermediate lobe.

### 3.5 Localization of HAP1 in neurosecretory elements of the posterior pituitary

In the posterior pituitary, HAP1 immunoreactivity was predominantly associated with neurosecretory structures rather than S100β-positive pituicytes. Double immunofluorescence analysis revealed no co-localization between HAP1 and the pituicyte marker S100β, indicating that HAP1 is not expressed in glial cells of the neurohypophysis (Figures 5A–D, Table 3). In contrast, strong co-localization of HAP1 was observed with both OXT- (Figures 5E–G) and AVP-immunoreactive neurosecretory structures (Figures 5I–K). HAP1-positive signals were frequently localized within enlarged, punctate, or ovoid OXT- and AVP-containing neurosecretory structures, putative Herring bodies. Quantitative analysis demonstrated that 76.68% of OXT-positive structures expressed HAP1, whereas 93.42% of HAP1-positive structures exhibited OXT immunoreactivity (Figure 5H, Table 3). Similarly, 71.53% of AVP-positive structures expressed HAP1, whereas 92.37% of HAP1-positive structures exhibited AVP immunoreactivity (Figure 5L, Table 3). These findings indicate that HAP1 is highly enriched in OXT- and AVP-containing neurosecretory structures of the posterior pituitary, putative Herring bodies, and is closely associated with oxytocinergic and vasopressinergic neurosecretory systems.

**Figure 5:**
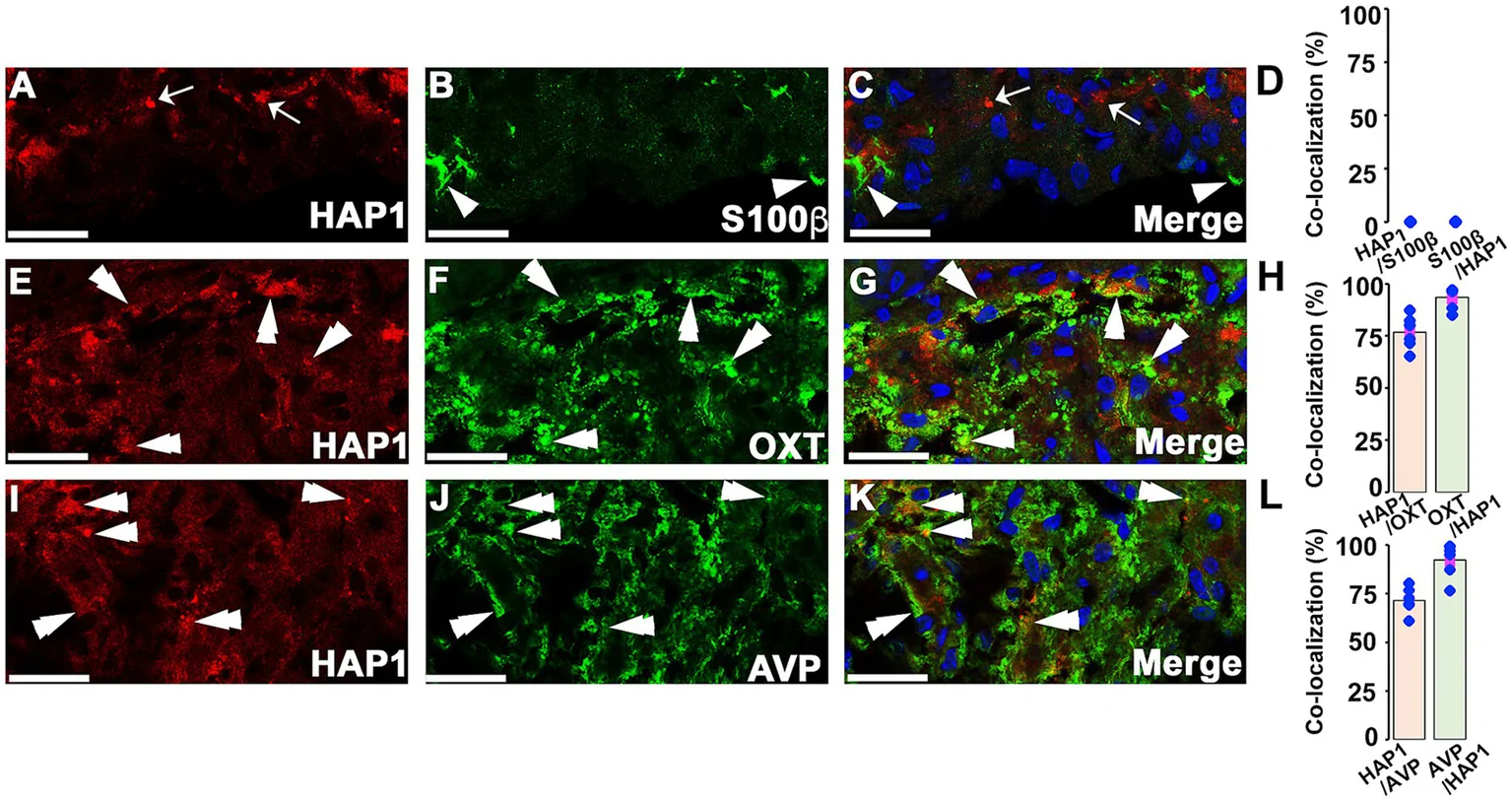
Double immunofluorescence showing HAP1 expression in relation to S100β-, OXT-, and AVP-immunoreactive structures in the mouse posterior pituitary **(A–C)** Posterior pituitary sections stained for HAP1 (red; **A**) and S100β (green; **B**). **(C)** Merged image showing no detectable co-localization of HAP1 and S100β **(D)** Quantitative analysis confirming the absence of reciprocal co-localization between HAP1 and S100β (HAP1/S100β and S100β/HAP1; 0.0% for both ratios) **(E–G)** Posterior pituitary sections stained for HAP1 (red; **E**) and OXT (green; **F**) **(G)** Merged image showing co-localization of HAP1 and OXT **(H)** Quantitative analysis showing the reciprocal co-localization ratios between HAP1 and OXT (HAP1/OXT and OXT/HAP1) **(I–K)** Posterior pituitary sections stained for HAP1 (red; **I**) and AVP (green; **J**) **(K)** Merged image showing co-localization of HAP1 and AVP **(L)** Quantitative analysis showing the reciprocal co-localization ratios between HAP1 and AVP (HAP1/AVP and AVP/HAP1). In **D**, **H**, and **L**, individual data points represent biological replicates (*n* = 6 mice), and bars indicate mean ± SEM. In **D**, all six individual values were 0%; consequently, the individual data points are superimposed at 0%. For each HAP1/marker pair, HAP1/marker represents the percentage of marker-positive structures that were HAP1-positive, whereas marker/HAP1 represents the percentage of HAP1-positive structures that were marker-positive within the corresponding double-label experiment. Reciprocal co-localization ratios were calculated independently for each HAP1/marker double-label experiment and therefore do not represent mutually exclusive fractions of a common HAP1-positive population. Arrows indicate single HAP1-positive structures, arrowheads indicate single S100β, OXT, or AVP-positive immunoreactivity, and double arrowheads indicate co-localization. Nuclei are counterstained with DAPI (blue). Scale bars = 25 μm. HAP1, Huntingtin-associated protein 1; OXT, oxytocin; AVP, arginine vasopressin.

### 3.6 Co-localization of HAP1 with OXT- and AVP-producing neurons in the PVN and SON of the hypothalamus

To determine whether the neurosecretory localization of HAP1 extends to its neuronal origins, we next examined its expression in magnocellular neurons of the PVN and SON. Consistent with the prominent localization of HAP1 in neurosecretory structures of the posterior pituitary (Figure 5), strong HAP1 immunoreactivity was observed in both the PVN and SON, which contain OXT- and AVP-producing magnocellular neurons (Figures 6A–C, 7A–C). Double immunofluorescence analysis demonstrated extensive co-localization of HAP1 with OXT-immunoreactive neurons in both the PVN (Figures 6D–F) and SON (Figures 6H–J). Likewise, extensive co-localization was observed between HAP1 and AVP-immunoreactive neurons in both the PVN (Figures 7D–F) and SON (Figures 7H–J).

**Figure 6:**
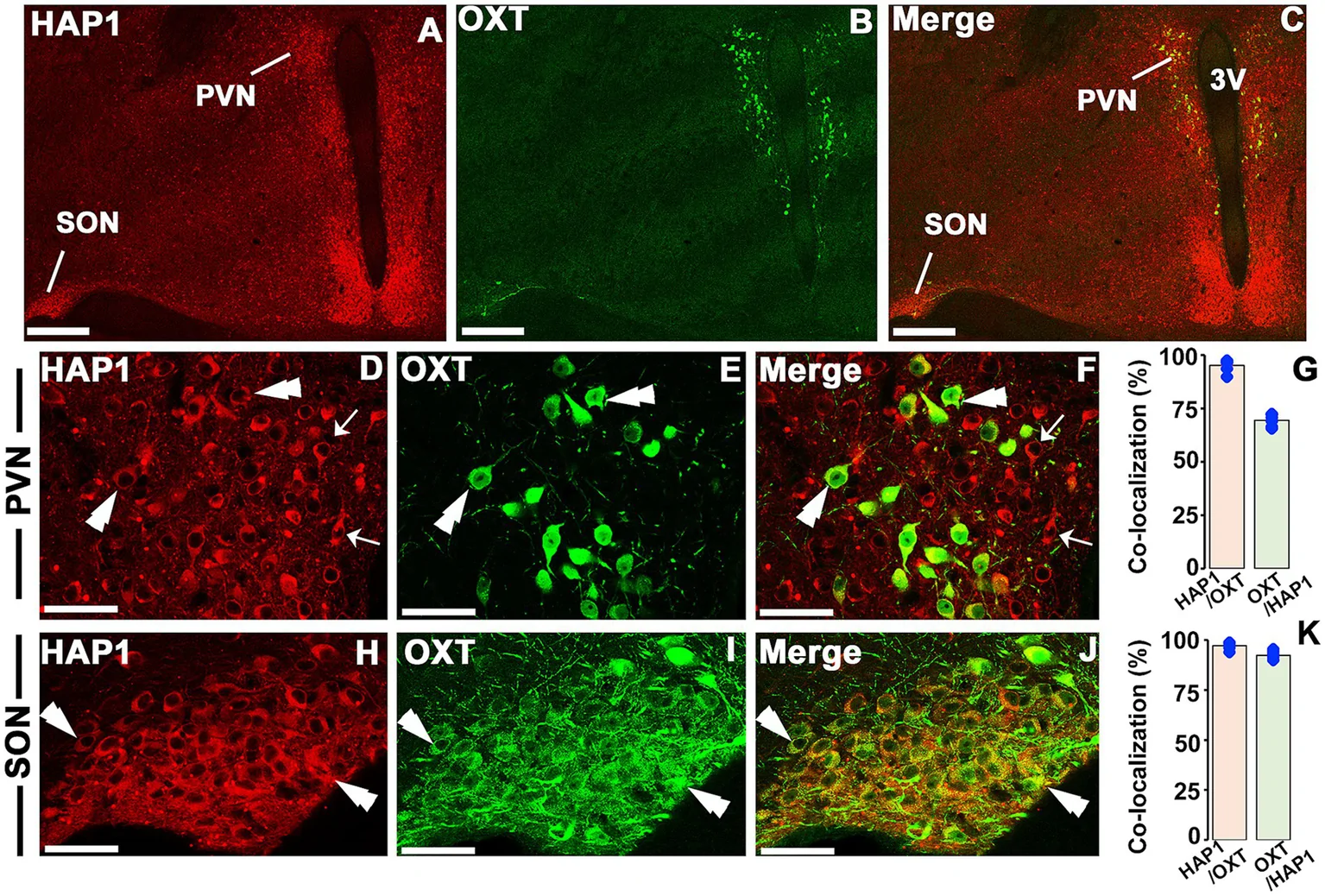
Double immunofluorescence staining of HAP1 with OXT-immunoreactive neurons in the hypothalamic paraventricular and supraoptic nuclei. **(A–C)** Low-magnification images showing the distribution of HAP1 (red; **A**) and OXT (green; **B**) in the hypothalamus. **(C)** Merged image showing the anatomical distribution of HAP1 and OXT in the PVN and SON. **(D–F)** Higher-magnification images of the PVN stained for HAP1 (red; **D**) and OXT (green; **E**). **(F)** Merged image showing co-localization of HAP1 and OXT. **(G)** Quantitative analysis showing the reciprocal co-localization ratios between HAP1 and OXT in the PVN (HAP1/OXT and OXT/HAP1). **(H–J)** Higher-magnification images of the SON stained for HAP1 (red; **H**) and OXT (green; **I**). **(J)** Merged image showing extensive co-localization of HAP1 and OXT. **(K)** Quantitative analysis showing the reciprocal co-localization ratios between HAP1 and OXT in the SON (HAP1/OXT and OXT/HAP1). In **G,K**, individual data points represent biological replicates (*n* = 6 mice), and bars indicate mean ± SEM. HAP1/OXT represents the percentage of OXT-positive neurons that were HAP1-positive, whereas OXT/HAP1 represents the percentage of HAP1-positive neurons that were OXT-positive within the corresponding HAP1/OXT double-label experiment. Arrows indicate single-labeled HAP1-positive neurons, arrowheads indicate single-labeled OXT-positive neurons, and double arrowheads indicate neurons co-labeled for HAP1 and OXT. 3 V, third ventricle; HAP1, huntingtin-associated protein 1; OXT, oxytocin; PVN, paraventricular nucleus; SON, supraoptic nucleus. Scale bars = 200 μm **(A–C)** and 50 μm **(D–F, H–J)**.

**Figure 7:**
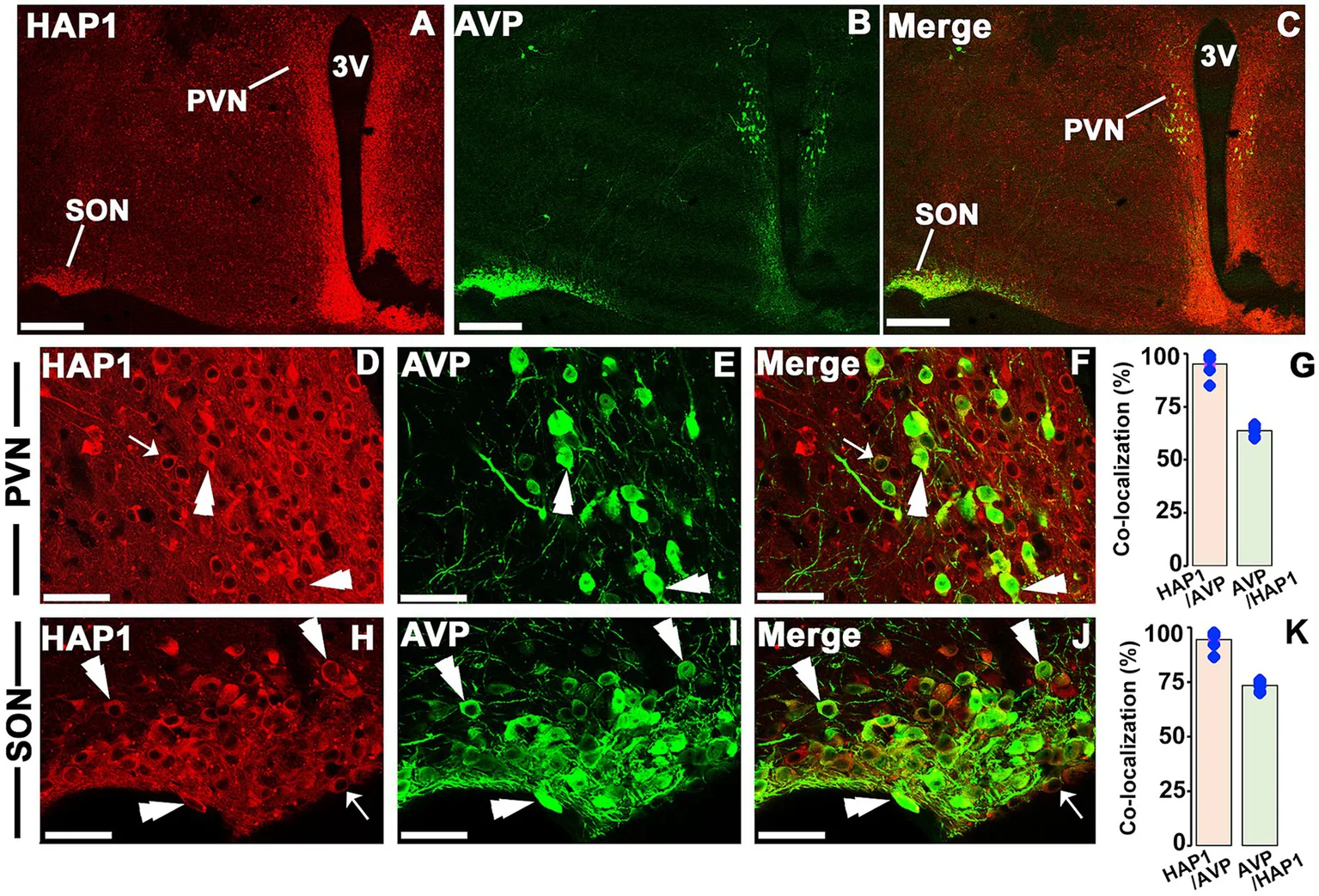
Double immunofluorescence staining of HAP1 with AVP-immunoreactive neurons in the hypothalamic paraventricular and supraoptic nuclei. **(A–C)** Low-magnification images showing the distribution of HAP1 (red; **A**) and AVP (green; **B**) in the hypothalamus. **(C)** Merged image showing the anatomical distribution of HAP1 and AVP in the PVN and SON. **(D–F)** Higher-magnification images of the PVN stained for HAP1 (red; **D**) and AVP (green; **E**). **(F)** Merged image showing co-localization of HAP1 and AVP. **(G)** Quantitative analysis showing the reciprocal co-localization ratios between HAP1 and AVP in the PVN (HAP1/AVP and AVP/HAP1). **(H–J)** Higher-magnification images of the SON stained for HAP1 (red; **H**) and AVP (green; **I**). **(J)** Merged image showing co-localization of HAP1 and AVP. **(K)** Quantitative analysis showing the reciprocal co-localization ratios between HAP1 and AVP in the SON (HAP1/AVP and AVP/HAP1). In **G,K**, individual data points represent biological replicates (*n* = 6 mice), and bars indicate mean ± SEM. HAP1/AVP represents the percentage of AVP-positive neurons that were HAP1-positive, whereas AVP/HAP1 represents the percentage of HAP1-positive neurons that were AVP-positive within the corresponding HAP1/AVP double-label experiment. Arrows indicate single-labeled HAP1-positive neurons, arrowheads indicate single-labeled AVP-positive neurons, and double arrowheads indicate neurons co-labeled for HAP1 and AVP. Scale bars = 200 μm in **A–C**; 50 μm in **D–F** and **H–J**. HAP1, huntingtin-associated protein 1; AVP, arginine vasopressin; PVN, paraventricular nucleus; SON, supraoptic nucleus.

Quantitative analysis confirmed that 95.23% of OXT-positive neurons in the PVN (Figure 6G, Table 3) and 97.29% in the SON (Figure 6K, Table 3) expressed HAP1. Similarly, 95.11% of AVP-positive neurons in the PVN (Figure 7G, Table 3) and 94.44% in the SON (Figure 7K, Table 3) co-expressed HAP1. Conversely, a substantial proportion of HAP1-positive neurons were identified as OXT-positive (69.53% in the PVN, Figure 6G; and 92.31% in the SON, Figure 6K, Table 3) or AVP-positive (63.78% in the PVN, Figure 7G; and 73.39% in the SON, Figure 7K, Table 3), indicating that HAP1 is highly enriched within these magnocellular neurosecretory populations.

Taken together with the findings in the posterior pituitary (Figure 5), these results demonstrate that HAP1 is strongly associated with OXT- and AVP-containing neurosecretory structures, putative Herring bodies, as well as with magnocellular OXT- and AVP-producing neurons in the PVN and SON. Collectively, these findings reveal a continuous and conserved enrichment of HAP1 throughout the magnocellular neurosecretory system, extending from neuronal cell bodies in the PVN and SON to axonal terminals in the posterior pituitary.

## 4 Discussion

The present study provides a comprehensive characterization of HAP1 expression across the hypothalamic–pituitary axis, revealing a highly cell-type-specific and compartment-dependent distribution pattern. By integrating Western blotting and immunohistochemistry with hormone-specific markers, our findings extend the anatomical characterization of HAP1 beyond neuronal systems to endocrine and neurosecretory cell populations. These distribution patterns provide an anatomical basis for considering potential roles of HAP1 in neuroendocrine regulation and cellular resilience (summarized in Figure 8), although these functions were not directly examined in the present study. Consistent with previous reports describing abundant HAP1 expression in the hypothalamus (Gutekunst et al., 1998; Fujinaga et al., 2004), we observed strong HAP1 immunoreactivity in hypothalamic neurons, including punctate cytoplasmic STBs. In contrast, the pituitary gland exhibited a distinct lobe-specific pattern, with moderate and scattered expression in the anterior lobe, stronger diffuse labeling in the intermediate lobe, and intense accumulation in the posterior lobe. The prominent HAP1 enrichment in the posterior pituitary is particularly notable, as this region represents a neurohemal interface composed primarily of hypothalamic axon terminals rather than intrinsic endocrine cells (Leng et al., 1999; Burbach et al., 2001).

**Figure 8:**
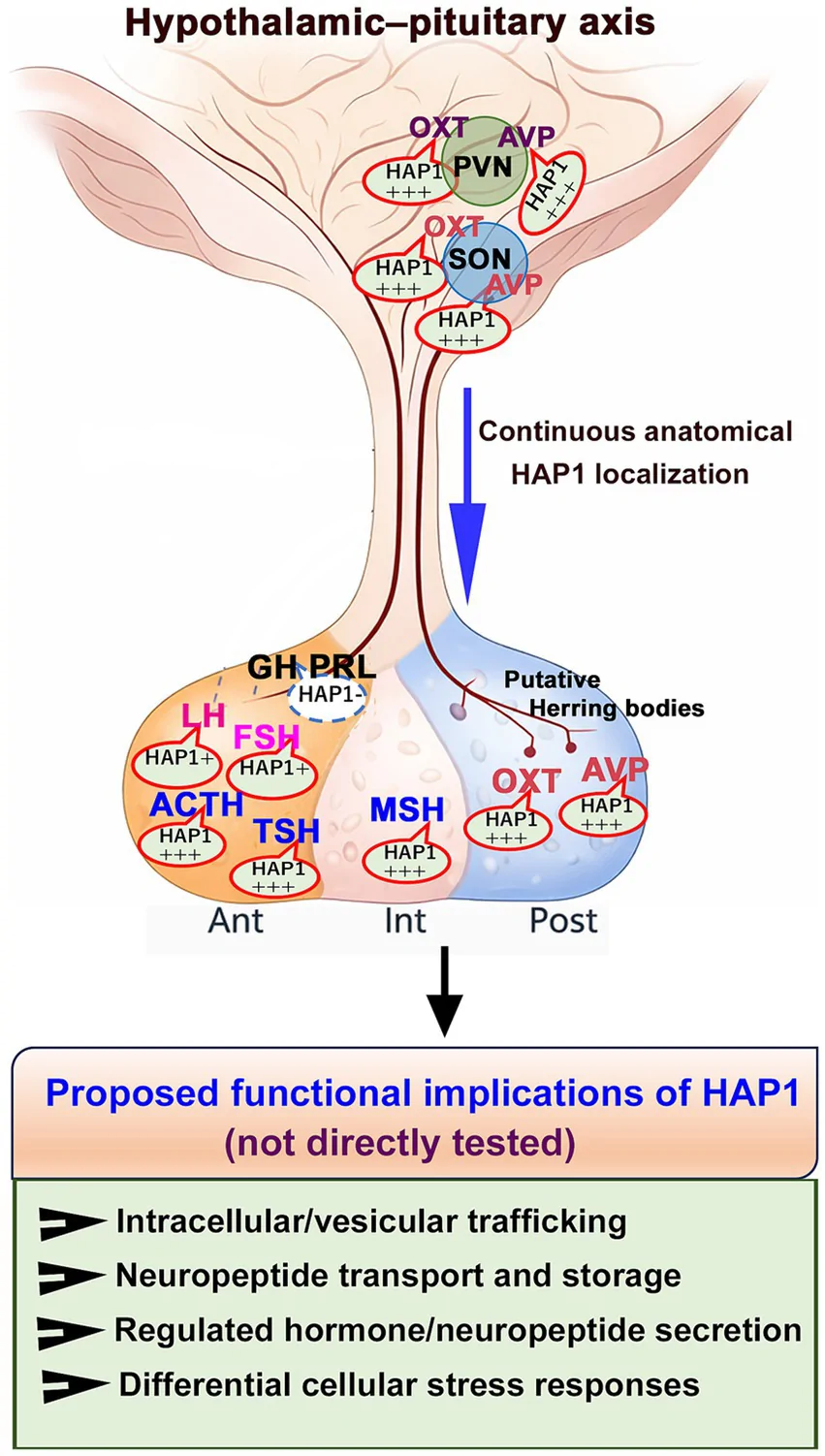
Schematic summary of the cell-type-specific distribution of HAP1 across the hypothalamic–pituitary axis and proposed functional implications. The upper schematic summarizes the anatomical findings demonstrated in the present study. HAP1 is highly expressed in magnocellular neurons of the hypothalamic paraventricular nucleus (PVN) and supraoptic nucleus (SON), where it extensively co-localizes with oxytocin (OXT)- and arginine vasopressin (AVP)-producing neurons. HAP1 expression extends continuously from hypothalamic neuronal cell bodies to OXT- and AVP-immunoreactive neurosecretory structures in the posterior (Post) pituitary, putative Herring bodies. In the anterior (Ant) and intermediate (Int) pituitary, HAP1 exhibits a cell-type-specific distribution, with high expression in corticotrophs (ACTH), thyrotrophs (TSH), and melanotrophs (MSH), limited expression in gonadotrophs (LH and FSH), and no detectable expression in somatotrophs (GH) or lactotrophs (PRL). The lower box summarizes proposed functional implications of these anatomical findings, including potential roles in intracellular/vesicular trafficking, neuropeptide transport and storage, regulated hormone/neuropeptide secretion, and differential cellular stress responses. These proposed functions were not directly examined in the present study and therefore represent hypotheses for future investigation rather than demonstrated effects of HAP1.

### 4.1 Cell-type-specific expression of HAP1 in the anterior and intermediate pituitary

A major finding of this study is the selective enrichment of HAP1 in specific anterior and intermediate pituitary cell types. High levels of HAP1 were observed in thyrotrophs, corticotrophs, and melanotrophs, whereas gonadotrophs exhibited relatively low expression, and somatotrophs and lactotrophs showed a complete absence of HAP1. This striking heterogeneity suggests that HAP1 expression is tightly linked to functional specialization among endocrine cell types. Thyrotrophs and corticotrophs are key regulators of metabolic and stress responses through the secretion of TSH and ACTH, respectively (Melmed, 2020). Similarly, melanotrophs in the intermediate lobe produce proopiomelanocortin-derived peptides, including MSH, which are involved in energy balance and neuroendocrine integration (Cone, 2005; Millington, 2007). Enrichment of HAP1 in these cell types raises the possibility that it may contribute to the intracellular processing, transport, or stimulus-dependent release of TSH, ACTH, and MSH. Such a role would be particularly relevant in endocrine cells that require tightly controlled peptide storage and rapid secretory responses to physiological demands. In addition, according to the previously proposed HAP1 protection hypothesis, cells with high HAP1 expression may exhibit increased resistance to cellular stress and neurodegeneration (Fujinaga et al., 2004, 2011; Takeshita et al., 2006, 2011; Islam et al., 2012, 2017, 2022, 2023, 2025; Meher et al., 2025a; Afrin et al., 2025). Accordingly, the enrichment of HAP1 in TSH-, ACTH-, and MSH-producing cells raises the hypothesis that HAP1 could contribute to cellular resilience in these endocrine populations. However, the present study did not examine cellular stress responses or survival, and therefore does not provide direct evidence for a protective effect of HAP1 in pituitary endocrine cells.

Previous studies have identified several mechanisms through which HAP1 may influence cellular function and survival. First, HAP1 is known to associate with intracellular trafficking machinery and stabilize vesicular transport, which may prevent the accumulation of misfolded proteins or dysfunctional vesicles within highly active secretory cells (Engelender et al., 1997; Block-Galarza et al., 1997; Li and Li, 2004). Second, HAP1 has been shown to regulate receptor trafficking and prevent lysosomal degradation of key signaling receptors, thereby sustaining trophic signaling pathways essential for cell survival (Rong et al., 2007; Wu et al., 2024). Third, HAP1-containing STBs may function as intracellular buffering or sequestration sites, reducing cellular stress by compartmentalizing potentially toxic proteins or aggregates (Takeshita et al., 2006, 2011; Fujinaga et al., 2007, 2009, 2011). These previously described mechanisms provide a conceptual basis for investigating whether HAP1 contributes to intracellular trafficking or cellular resilience in pituitary endocrine cells. However, whether these mechanisms operate in the endocrine cell populations identified in the present study remains to be determined experimentally.

### 4.2 Absence of detectable HAP1 in somatotrophs and lactotrophs: potential functional implications

It is intriguing to note that functional alterations of somatotroph and lactotroph activity have been reported in several NDs, largely as a consequence of disrupted hypothalamic regulation of endocrine axes (Saleh et al., 2009; Aziz et al., 2007; Newhouse and Chemali, 2020). Although these disorders do not typically involve overt degeneration of pituitary cells, they are associated with impaired growth hormone and prolactin dynamics. In this context, the absence of detectable HAP1 in somatotrophs and lactotrophs observed in the present study is noteworthy and further emphasizes the cell-type-specific distribution of HAP1 within the anterior pituitary. However, the present anatomical findings do not establish that the absence of HAP1 confers greater functional or cellular vulnerability. Whether HAP1-negative and HAP1-enriched endocrine populations differ in their responses to cellular stress or pathological conditions will require direct experimental investigation.

### 4.3 HAP1 in neurosecretory structures: implications for vesicle trafficking and hormone release

One of the most significant findings of this study is the strong co-localization of HAP1 with OXT- and AVP-immunoreactive enlarged punctate structures, putative Herring bodies, indicating that HAP1 is enriched in these neurosecretory compartments of the posterior pituitary. These enlarged neurosecretory structures serve as sites of storage and regulated release of OXT and AVP (Burbach et al., 2001). The absence of co-localization with the glial marker S100β further confirms that HAP1 is restricted to neuronal elements rather than pituicytes. The enrichment of HAP1 in these structures provides an anatomical association between HAP1 and the neurosecretory apparatus but does not directly demonstrate a role in vesicle trafficking, storage, or exocytosis. Given that HAP1 interacts with microtubule-associated transport systems and vesicular trafficking (Engelender et al., 1997; Li and Li, 2004), the present localization pattern raises the hypothesis that HAP1 may participate in the trafficking or stabilization of neurosecretory vesicles within the hypothalamic–neurohypophyseal system. This possibility requires direct functional investigation.

From a physiological perspective, AVP plays a central role in water homeostasis and blood pressure regulation, while OXT is involved in reproductive functions such as parturition and lactation, as well as social and stress-related behaviors (Neumann and Landgraf, 2012). The precise temporal release of these neuropeptides is critical for maintaining systemic homeostasis. In particular, AVP regulates renal water reabsorption and urine concentration (Bankir et al., 2017), and emerging evidence indicates that OXT can modulate bladder function and micturition reflexes through central and peripheral mechanisms (Cafarchio et al., 2020). Thus, the localization of HAP1 within AVP- and OXT-immunoreactive neurosecretory structures establishes an anatomical association with neuropeptide systems involved in fluid balance, reproductive functions, and lower urinary tract physiology. However, the present study does not demonstrate that HAP1 itself regulates hormone release or these downstream physiological functions.

An important consideration in interpreting the reciprocal co-localization ratios is that each HAP1/marker combination was examined in an independent double-label experiment. Thus, the high OXT/HAP1 and AVP/HAP1 ratios observed in the posterior pituitary indicate that HAP1 is extensively localized within both OXT-containing and AVP-containing neurosecretory structures when these populations are examined independently; they do not indicate that individual HAP1-positive structures necessarily contain both neuropeptides. Similarly, the reciprocal ratios obtained for different anterior pituitary hormones represent marker-specific associations with HAP1 rather than mutually exclusive subdivisions of a common HAP1-positive population. Because simultaneous labeling of different endocrine hormones or of OXT and AVP was not performed, the present study cannot determine the extent of multi-marker co-expression or whether individual neurosecretory swellings contain both OXT and AVP. Accordingly, our findings are interpreted as demonstrating HAP1 localization within independently identified endocrine and neurosecretory populations rather than co-expression among the different hormonal markers.

### 4.4 Anatomical continuity of HAP1 along the hypothalamic–neurohypophyseal axis

Our current findings further demonstrate that HAP1 is highly expressed in magnocellular neurons of both the PVN and SON, where it shows extensive co-localization with OXT- and AVP-immunoreactive neurons. When considered together with the posterior pituitary findings, these observations reveal a striking anatomical continuity of HAP1 localization from hypothalamic magnocellular neuronal cell bodies to neurosecretory structures in the posterior pituitary. This anatomical pattern is consistent with, but does not directly demonstrate, a role for HAP1 in intracellular trafficking or axonal transport. Previous studies implicating HAP1 in vesicle motility and cargo transport in neurons (Li and Li, 2004; McGuire et al., 2006; Yuen et al., 2012) provide a mechanistic basis for investigating whether HAP1 contributes to neurosecretory vesicle transport in magnocellular neurons. Functional studies will be required to determine whether HAP1 influences vesicle trafficking, neuropeptide release, or cellular resilience within the hypothalamic–neurohypophyseal system.

### 4.5 Limitations and future directions

While the present study provides a detailed anatomical and immunohistochemical characterization of HAP1 in the hypothalamic–pituitary axis, several limitations should be acknowledged. Most importantly, the study is observational, and no functional manipulation of HAP1 was performed. Therefore, the proposed roles of HAP1 in intracellular trafficking, hormone secretion, and cellular resilience should be regarded as hypotheses based on the present localization data together with previously reported functions of HAP1. Future studies employing genetic manipulation and functional assays will be necessary to test these possibilities directly. Second, although strong associations with OXT- and AVP-containing systems were observed, whether HAP1 contributes to neuropeptide transport and the molecular mechanisms potentially involved remain unclear and warrant further investigation. Importantly, although OXT and AVP immunoreactivity were used to identify enlarged punctate neurosecretory compartments, the precise ultrastructural localization of HAP1 within the posterior pituitary was not confirmed. Future studies using electron microscopy will be necessary to definitively establish the ultrastructural identity and subcellular localization of these HAP1-immunoreactive neurosecretory structures and to determine whether they correspond to Herring bodies. Another limitation is the exclusive use of male mice. Although previous studies have not established a clear sex-dependent difference in HAP1 distribution, this cannot be assumed for the hypothalamic–pituitary axis, where endocrine and neurosecretory systems are strongly influenced by sex steroids and reproductive hormonal status. Thus, sex and hormonal state may influence HAP1 expression, cellular distribution, or its association with specific endocrine and neurosecretory populations. Future studies directly comparing male and female mice, including females at defined stages of the estrous cycle, will be necessary to determine whether HAP1 distribution within the hypothalamic–pituitary axis exhibits sex- or hormonal-state-dependent variation. An additional limitation concerns the interpretation of the reciprocal co-localization ratios. Because each HAP1/marker combination was examined in a separate double-label experiment, the same individual HAP1-positive cells, neurons, or neurosecretory structures were not simultaneously phenotyped for multiple markers. Consequently, sums of marker/HAP1 ratios exceeding 100% may be compatible with biological overlap among marker-defined populations, but the present pairwise double-label experiments cannot establish such overlap. Variability arising from independent tissue sampling and staining experiments may also contribute to these values. Furthermore, although the cell-specific antibodies produced the expected anatomical staining patterns and have been used in previous studies, independent specificity controls were not performed for every marker antibody in the present study. Additional antibody-validation approaches, together with simultaneous triple or multiplex immunofluorescence, would help further clarify the extent to which these numerical findings reflect biological co-localization among marker-defined populations. Finally, given the previously proposed neuroprotective functions of HAP1, it will be important to determine whether endocrine dysfunction contributes to disease pathophysiology in conditions associated with altered HAP1 expression.

## 5 Conclusion

The present study identifies three major features of HAP1 distribution within the hypothalamic–pituitary axis: cell-type-specific expression among endocrine cell populations, absence of detectable HAP1 from somatotrophs and lactotrophs, and continuous anatomical localization throughout the magnocellular hypothalamic–neurohypophyseal system. HAP1 was enriched in thyrotrophs, corticotrophs, melanotrophs, magnocellular neurons, and neurosecretory structures, revealing a previously unrecognized cell-type-specific distribution within the hypothalamic–pituitary axis. The continuous localization of HAP1 from hypothalamic neuronal somata to posterior pituitary neurosecretory structures provides an anatomical basis for the hypothesis that HAP1 may participate in neuropeptide trafficking or regulated secretion, although these functions were not directly examined in the present study. Collectively, these findings extend the anatomical characterization of HAP1 into the neuroendocrine system and provide a foundation for future functional studies examining its potential roles in intracellular trafficking, hormone secretion, and cellular stress responses.

## Data Availability

The raw data supporting the conclusions of this article will be made available by the authors, without undue reservation.

## Glossary

3 V: 3rd ventricle
ACTH: Adrenocorticotropic hormone
AVP: Arginine vasopressin
DAB: Diaminobenzidine
ECL: Enhanced chemiluminescence
FSH: Follicle-stimulating hormone
GH: Growth hormone
HAP1: Huntingtin-associated protein 1
LH: Luteinizing hormone
MMB: Medetomidine, midazolam, and butorphanol
MSH: Melanocyte-stimulating hormone
NDS: Normal donkey serum
OXT: Oxytocin
PB: Phosphate buffer
PBS: Phosphate-buffered saline
PRL: Prolactin
PVN: Paraventricular nucleus
RRID: research resource identifier
SEM: Standard error of the mean
SON: Supraoptic nucleus
SPSS: Statistical Package for the Social Sciences
STB: Stigmoid body
PBST: phosphate-buffered saline containing Tween 20
TSH: Thyroid-stimulating hormone

## References

[ref1] Afrin M. Islam M. N. Meher M. M. Jahan M. R. Nozaki K. Masumoto K. . (2025). Neuroanatomical mapping of huntingtin-associated protein 1 across the rostral and caudal clusters of mouse raphe nuclei and its immunohistochemical relationships with serotonin. Front. Neuroanat. 19:1625793. doi: 10.3389/fnana.2025.1625793, 40766770 PMC12321798

[ref2] Aziz N. A. Swaab D. F. Pijl H. Roos R. A. (2007). Hypothalamic dysfunction and neuroendocrine and metabolic alterations in Huntington's disease: clinical consequences and therapeutic implications. Rev. Neurosci. 18, 223–251. doi: 10.1515/revneuro.2007.18.3-4.223, 18019608

[ref3] Bankir L. Bichet D. G. Morgenthaler N. G. (2017). Vasopressin: physiology, assessment and osmosensation. J. Intern. Med. 282, 284–297. doi: 10.1111/joim.12645, 28649750

[ref4] Block-Galarza J. Chase K. O. Sapp E. Vaughn K. T. Vallee R. B. DiFiglia M. . (1997). Fast transport and retrograde movement of huntingtin and HAP 1 in axons. Neuroreport 8, 2247–2250. doi: 10.1097/00001756-199707070-00031, 9243620

[ref5] Burbach J. P. Luckman S. M. Murphy D. Gainer H. (2001). Gene regulation in the magnocellular hypothalamo-neurohypophysial system. Physiol. Rev. 81, 1197–1267. doi: 10.1152/physrev.2001.81.3.1197, 11427695

[ref6] Cafarchio E. M. da Silva L. A. Auresco L. C. Rodart I. F. de Souza J. S. Antonio B. B. . (2020). Oxytocin reduces Intravesical pressure in anesthetized female rats: action on oxytocin receptors of the urinary bladder. Front. Physiol. 11:382. doi: 10.3389/fphys.2020.00382, 32435202 PMC7218109

[ref7] Callewaere C. Banisadr G. Desarménien M. G. Mechighel P. Kitabgi P. Rostène W. H. . (2006). The chemokine SDF-1/CXCL12 modulates the firing pattern of vasopressin neurons and counteracts induced vasopressin release through CXCR4. Proc. Natl. Acad. Sci. USA 103, 8221–8226. doi: 10.1073/pnas.0602620103, 16702540 PMC1570101

[ref8] Chen X. Xin N. Pan Y. Zhu L. Yin P. Liu Q. . (2020). Huntingtin-associated protein 1 in mouse hypothalamus stabilizes glucocorticoid receptor in stress response. Front. Cell. Neurosci. 14:125. doi: 10.3389/fncel.2020.00125, 32581713 PMC7289054

[ref9] Cone R. D. (2005). Anatomy and regulation of the central melanocortin system. Nat. Neurosci. 8, 571–578. doi: 10.1038/nn1455, 15856065

[ref10] Dayanithi G. Forostyak O. Forostyak S. Kayano T. Ueta Y. Verkhratsky A. (2018). Vasopressin and oxytocin in sensory neurones: expression, exocytotic release and regulation by lactation. Sci. Rep. 8:13084. doi: 10.1038/s41598-018-31361-1, 30166555 PMC6117293

[ref11] Engelender S. Sharp A. H. Colomer V. Tokito M. K. Lanahan A. Worley P. . (1997). Huntingtin-associated protein 1 (HAP1) interacts with the p150Glued subunit of dynactin. Hum. Mol. Genet. 6, 2205–2212. doi: 10.1093/hmg/6.13.2205, 9361024

[ref12] Fujinaga R. Kawano J. Matsuzaki Y. Kamei K. Yanai A. Sheng Z. . (2004). Neuroanatomical distribution of huntingtin-associated protein 1-mRNA in the male mouse brain. J. Comp. Neurol. 478, 88–109. doi: 10.1002/cne.20277 15334651

[ref13] Fujinaga R. Takeshita Y. Uozumi K. Yanai A. Yoshioka K. Kokubu K. . (2009). Microtubule-dependent formation of the stigmoid body as a cytoplasmic inclusion distinct from pathological aggresomes. Histochem. Cell Biol. 132, 305–318. doi: 10.1007/s00418-009-0618-9, 19578869

[ref14] Fujinaga R. Takeshita Y. Yoshioka K. Nakamura H. Shinoda S. Islam M. N. . (2011). Intracellular colocalization of HAP1/STBs with steroid hormone receptors and its enhancement by a proteasome inhibitor. Exp. Cell Res. 317, 1689–1700. doi: 10.1016/j.yexcr.2011.05.004, 21609716

[ref15] Fujinaga R. Yanai A. Nakatsuka H. Yoshida K. Takeshita Y. Uozumi K. . (2007). Anti-human placental antigen complex X-P2 (hPAX-P2) anti-serum recognizes C-terminus of huntingtin-associated protein 1A common to 1B as a determinant marker for the stigmoid body. Histochem. Cell Biol. 128, 335–348. doi: 10.1007/s00418-007-0315-5, 17687563

[ref16] Gutekunst C. A. Li S. H. Yi H. Ferrante R. J. Li X. J. Hersch S. M. (1998). The cellular and subcellular localization of huntingtin-associated protein 1 (HAP1): comparison with huntingtin in rat and human. J. Neurosci. 18, 7674–7686. doi: 10.1523/JNEUROSCI.18-19-07674.1998, 9742138 PMC6793025

[ref17] Gutekunst C. A. Li S. H. Yi H. Mulroy J. S. Kuemmerle S. Jones R. . (1999). Nuclear and neuropil aggregates in Huntington's disease: relationship to neuropathology. J. Neurosci. 19, 2522–2534. doi: 10.1523/JNEUROSCI.19-07-02522.1999, 10087066 PMC6786077

[ref18] Hidema S. Sato K. Mizukami H. Takahashi Y. Maejima Y. Shimomura K. . (2024). Oxytocin receptor-expressing neurons in the medial preoptic area are essential for lactation, whereas those in the lateral septum are not critical for maternal behavior. Neuroendocrinology 114, 517–537. doi: 10.1159/000535362, 38071956 PMC11151981

[ref19] Huang Q. H. Zhao G. K. Wang H. Q. Wei F. H. Zhang J. Y. Zhang J. B. . (2024). Single-cell transcriptional profile construction of rat pituitary glands before and after sexual maturation and identification of novel marker Spp1 in Gonadotropes. Int. J. Mol. Sci. 25:4694. doi: 10.3390/ijms25094694, 38731915 PMC11083676

[ref20] Iremonger K. J. Power E. M. (2025). The paraventricular nucleus of the hypothalamus: a key node in the control of behavioural states. J. Physiol. 603, 2231–2243. doi: 10.1113/JP288366, 40119815 PMC12013795

[ref21] Islam M. N. Fujinaga R. Yanai A. Jahan M. R. Takeshita Y. Kokubu K. . (2012). Characterization of the “sporadically lurking HAP1-immunoreactive (SLH) cells” in the hippocampus, with special reference to the expression of steroid receptors, GABA, and progenitor cell markers. Neuroscience 210, 67–81. doi: 10.1016/j.neuroscience.2012.02.029, 22421101

[ref22] Islam M. N. Maeda N. Miyasato E. Jahan M. R. Tarif A. M. M. Ishino T. . (2020a). Expression of huntingtin-associated protein 1 in adult mouse dorsal root ganglia and its neurochemical characterization in reference to sensory neuron subpopulations. IBRO Reports 9, 258–269. doi: 10.1016/j.ibror.2020.10.001, 33089002 PMC7560692

[ref23] Islam M. N. Maruyama M. Jahan M. R. Afrin M. Meher M. M. Nozaki K. . (2025). Neuroanatomical distribution of endogenous huntingtin and its immunohistochemical relationships with STB/HAP1 in the adult mouse brain and spinal cord. Neurosci. Res. 213, 1–22. doi: 10.1016/j.neures.2025.01.003, 39832698

[ref24] Islam M. N. Miyasato E. Jahan M. R. Tarif A. M. M. Nozaki K. Masumoto K. h. . (2022). Mapping of STB/HAP1 immunoreactivity in the mouse brainstem and its relationships with choline acetyltransferase, with special emphasis on cranial nerve motor and preganglionic autonomic nuclei. Neuroscience 499, 40–63. doi: 10.1016/j.neuroscience.2022.07.016 35870563

[ref25] Islam M. N. Sakimoto Y. Jahan M. R. Ishida M. Tarif A. M. M. Nozaki K. . (2020b). Androgen affects the dynamics of intrinsic plasticity of pyramidal neurons in the CA1 hippocampal subfield in adolescent male rats. Neuroscience 440, 15–29. doi: 10.1016/j.neuroscience.2020.05.025, 32450298

[ref26] Islam M. N. Sakimoto Y. Jahan M. R. Miyasato E. Tarif A. M. M. Nozaki K. . (2021). Androgen affects the inhibitory avoidance memory by primarily acting on androgen receptor in the brain in adolescent male rats. Brain Sci. 11:239. doi: 10.3390/brainsci11020239, 33672867 PMC7918178

[ref27] Islam M. N. Sakurai Y. Hiwaki Y. Tarif A. M. M. Afrin M. Meher M. M. . (2023). Immunohistochemical distribution and neurochemical characterization of huntingtin-associated protein 1 Immunoreactive neurons in the adult mouse lingual ganglia. Brain Sci. 13:258. doi: 10.3390/brainsci13020258, 36831801 PMC9954002

[ref28] Islam M. N. Takeshita Y. Yanai A. Imagawa A. Jahan M. R. Wroblewski G. . (2017). Immunohistochemical analysis of huntingtin-associated protein 1 in adult rat spinal cord and its regional relationship with androgen receptor. Neuroscience 340, 201–217. doi: 10.1016/j.neuroscience.2016.10.053, 27984179

[ref29] Jahan M. R. Islam M. N. Yanai A. Shinoda K. (2025). Differential distribution of sex hormone receptors in the subparaventricular zone and suprachiasmatic nucleus of adult and early postnatal mouse brain. Brain Mech. 148–150:202521. doi: 10.1016/j.bramec.2025.202521

[ref30] Jahan M. R. Kokubu K. Islam M. N. Matsuo C. Yanai A. Wroblewski G. . (2015). Species differences in androgen receptor expression in the medial preoptic and anterior hypothalamic areas of adult male and female rodents. Neuroscience 284, 943–961. doi: 10.1016/j.neuroscience.2014.11.003, 25446364

[ref31] Kamei K. Matsuzaki Y. Hanada K. Nagano M. Nakahama K. Shinoda K. (2001). Distribution of HAP1 mRNA in the rat brain. Acta Anat. Nippon. 76:A-91.

[ref32] Kawabe T. Kawabe K. Sapru H. N. (2012). Effect of barodenervation on cardiovascular responses elicited from the hypothalamic arcuate nucleus of the rat. PLoS One 7:e53111. doi: 10.1371/journal.pone.0053111, 23300873 PMC3531379

[ref33] Kestell G. R. Anderson R. L. Clarke J. N. Haberberger R. V. Gibbins I. L. (2015). Primary afferent neurons containing calcitonin gene-related peptide but not substance P in forepaw skin, dorsal root ganglia, and spinal cord of mice. J. Comp. Neurol. 523, 2555–2569. doi: 10.1002/cne.23804, 26010480

[ref34] Lee K. Y. Rhodes J. S. Saif M. T. A. (2023). Astrocyte-mediated transduction of muscle Fiber contractions synchronizes hippocampal neuronal network development. Neuroscience 515, 25–36. doi: 10.1016/j.neuroscience.2023.01.028, 36736611 PMC10023357

[ref35] Leng G. Brown C. H. Russell J. A. (1999). Physiological pathways regulating the activity of magnocellular neurosecretory cells. Prog. Neurobiol. 57, 625–655. doi: 10.1016/s0301-0082(98)00072-0, 10221785

[ref36] Li S. H. Gutekunst C. A. Hersch S. M. Li X. J. (1998). Association of HAP1 isoforms with a unique cytoplasmic structure. J. Neurochem. 71, 2178–2185. doi: 10.1046/j.1471-4159.1998.71052178.x, 9798945

[ref37] Li S. H. Li X. J. (2004). Huntingtin-protein interactions and the pathogenesis of Huntington's disease. Trends Genet. 20, 146–154. doi: 10.1016/j.tig.2004.01.008, 15036808

[ref38] Li X. J. Li S. H. Sharp A. H. Nucifora F. C. Jr. Schilling G. Lanahan A. . (1995). A huntingtin-associated protein enriched in brain with implications for pathology. Nature 378, 398–402. doi: 10.1038/378398a0, 7477378

[ref39] Li S. H. Yu Z. X. Li C. L. Nguyen H. P. Zhou Y. X. Deng C. . (2003). Lack of huntingtin-associated protein-1 causes neuronal death resembling hypothalamic degeneration in Huntington's disease. J. Neurosci. 23, 6956–6964. doi: 10.1523/JNEUROSCI.23-17-06956.2003, 12890790 PMC6740731

[ref40] Liao M. Chen X. Han J. Yang S. Peng T. Li H. (2010). Selective expression of huntingtin-associated protein 1 in {beta}-cells of the rat pancreatic islets. J. Histochem. Cytochem. 58, 255–263. doi: 10.1369/jhc.2009.954479, 19901268 PMC2825491

[ref41] Liao M. Shen J. Zhang Y. Li S. H. Li X. J. Li H. (2005). Immunohistochemical localization of huntingtin-associated protein 1 in endocrine system of the rat. J. Histochem. Cytochem. 53, 1517–1524. doi: 10.1369/jhc.5A6662.2005, 16087704 PMC3957544

[ref42] Lombardero M. Quintanar-Stephano A. Rotondo F. Horvath E. Kovacs K. (2020). Effect of thyroxin on cell morphology and hormone secretion of pituitary grafts in rats. Ann. Anat. 230:151486. doi: 10.1016/j.aanat.2020.151486, 32145383

[ref43] Mackenzie K. D. Duffield M. D. Peiris H. Phillips L. Zanin M. P. Teo E. H. . (2014). Huntingtin-associated protein 1 regulates exocytosis, vesicle docking, readily releasable pool size and fusion pore stability in mouse chromaffin cells. J. Physiol. 592, 1505–1518. doi: 10.1113/jphysiol.2013.268342, 24366265 PMC3979608

[ref44] McGuire J. R. Rong J. Li S. H. Li X. J. (2006). Interaction of huntingtin-associated protein-1 with kinesin light chain: implications in intracellular trafficking in neurons. J. Biol. Chem. 281, 3552–3559. doi: 10.1074/jbc.M509806200, 16339760

[ref45] Meher M. M. Afrin M. Jahan M. R. Nozaki K. Masumoto K. Yanai A. . (2025b). Neurochemical heterogeneity of ChAT-immunoreactive neurons in the basal forebrain cholinergic nuclei and striatum in reference to CGRP, CCK, and calcium-binding proteins. Acta Histochem. 127:152291. doi: 10.1016/j.acthis.2025.152291, 40925258

[ref46] Meher M. M. Islam M. N. Yanai A. Afrin M. Jahan M. R. Nozaki K. . (2025a). Immunohistochemical relationships of huntingtin-associated protein 1 with choline acetyltransferase in the forebrain cholinergic nuclei of adult mice. Cell Tissue Res. 401, 237–263. doi: 10.1007/s00441-025-03996-w, 40682600

[ref47] Melmed S. (2020). Pituitary-tumor Endocrinopathies. N. Engl. J. Med. 382, 937–950. doi: 10.1056/NEJMra1810772, 32130815

[ref48] Metzger S. Rong J. Nguyen H. P. Cape A. Tomiuk J. Soehn A. S. . (2008). Huntingtin-associated protein-1 is a modifier of the age-at-onset of Huntington's disease. Hum. Mol. Genet. 17, 1137–1146. doi: 10.1093/hmg/ddn003, 18192679

[ref49] Millington G. W. (2007). The role of proopiomelanocortin (POMC) neurones in feeding behaviour. Nutr. Metab. (Lond.) 4:18. doi: 10.1186/1743-7075-4-18, 17764572 PMC2018708

[ref50] Nagano M. Shinoda K. (1994). Coexistence of the stigmoid body and estrogen receptor in some neuronal groups involved in rat reproductive functions. Brain Res. 634, 296–304. doi: 10.1016/0006-8993(94)91933-x, 8131080

[ref51] Neumann I. D. Landgraf R. (2012). Balance of brain oxytocin and vasopressin: implications for anxiety, depression, and social behaviors. Trends Neurosci. 35, 649–659. doi: 10.1016/j.tins.2012.08.004, 22974560

[ref52] Newhouse A. Chemali Z. (2020). Neuroendocrine disturbances in neurodegenerative disorders: a scoping review. Psychosomatics 61, 105–115. doi: 10.1016/j.psym.2019.11.002, 31918850

[ref53] Nishimura H. Kawasaki M. Matsuura T. Suzuki H. Motojima Y. Baba K. . (2020). Acute mono-arthritis activates the Neurohypophysial system and Hypothalamo-pituitary adrenal Axis in rats. Front. Endocrinol. (Lausanne) 11:43. doi: 10.3389/fendo.2020.00043, 32117068 PMC7026388

[ref54] Nishimura N. Yonemoto J. Miyabara Y. Sato M. Tohyama C. (2003). Rat thyroid hyperplasia induced by gestational and lactational exposure to 2,3,7,8-tetrachlorodibenzo-p-dioxin. Endocrinology 144, 2075–2083. doi: 10.1210/en.2002-220737, 12697716

[ref55] Oka S. Shiraishi K. Fujimoto M. Katiyar A. Takii R. Nakai A. . (2017). Role of heat shock factor 1 in conserving cholesterol transportation in Leydig cell steroidogenesis via steroidogenic acute regulatory protein. Endocrinology 158, 2648–2658. doi: 10.1210/en.2017-00132, 28575284

[ref56] Park S. H. Jang J. H. Lee Y. M. Kim J. S. Kim K. H. Kim Y. Z. (2017). Function of cell-cycle regulators in predicting silent pituitary adenoma progression following surgical resection. Oncol. Lett. 14, 7121–7130. doi: 10.3892/ol.2017.7117, 29344143 PMC5754893

[ref57] Rong J. Li S. H. Li X. J. (2007). Regulation of intracellular HAP1 trafficking. J. Neurosci. Res. 85, 3025–3029. doi: 10.1002/jnr.21326, 17474105

[ref58] Sakai Y. Hosaka M. Hira Y. Harumi T. Ohsawa Y. Wang H. . (2003). Immunocytochemical localization of secretogranin III in the anterior lobe of male rat pituitary glands. J. Histochem. Cytochem. 51, 227–238. doi: 10.1177/002215540305100211, 12533531

[ref59] Saleh N. Moutereau S. Durr A. Krystkowiak P. Azulay J. P. Tranchant C. . (2009). Neuroendocrine disturbances in Huntington's disease. PLoS One 4:e4962. doi: 10.1371/journal.pone.0004962, 19319184 PMC2655649

[ref60] Sheng J. A. Bales N. J. Myers S. A. Bautista A. I. Roueinfar M. Hale T. M. . (2021). The hypothalamic-pituitary-adrenal Axis: development, programming actions of hormones, and maternal-fetal interactions. Front. Behav. Neurosci. 14:601939. doi: 10.3389/fnbeh.2020.601939, 33519393 PMC7838595

[ref61] Sheng G. Chang G. Q. Lin J. Y. Yu Z. X. Fang Z. H. Rong J. . (2006). Hypothalamic huntingtin-associated protein 1 as a mediator of feeding behavior. Nat. Med. 12, 526–533. doi: 10.1038/nm1382, 16604089

[ref62] Sheng G. Xu X. Lin Y. F. Wang C. E. Rong J. Cheng D. . (2008). Huntingtin-associated protein 1 interacts with Ahi1 to regulate cerebellar and brainstem development in mice. J. Clin. Invest. 118, 2785–2795. doi: 10.1172/JCI35339, 18636121 PMC2467384

[ref63] Shinoda K. Mori S. Ohtsuki T. Osawa Y. (1992). An aromatase-associated cytoplasmic inclusion, the "stigmoid body," in the rat brain: I. Distribution in the forebrain. J. Comp. Neurol. 322, 360–376. doi: 10.1002/cne.903220306, 1325485

[ref64] Shinoda K. Nagano M. Osawa Y. (1993). An aromatase-associated cytoplasmic inclusion, the "stigmoid body," in the rat brain: II. Ultrastructure (with a review of its history and nomenclature). J. Comp. Neurol. 329, 1–19. doi: 10.1002/cne.903290102, 8384220

[ref65] Takeshita Y. Fujinaga R. Kokubu K. Islam M. N. Jahan M. R. Yanai A. . (2011). Interaction of ataxin-3 with huntingtin-associated protein 1 through Josephin domain. Neuroreport 22, 232–238. doi: 10.1097/WNR.0b013e32834505f4, 21386698

[ref66] Takeshita Y. Fujinaga R. Zhao C. Yanai A. Shinoda K. (2006). Huntingtin-associated protein 1 (HAP1) interacts with androgen receptor (AR) and suppresses SBMA-mutant-AR-induced apoptosis. Hum. Mol. Genet. 15, 2298–2312. doi: 10.1093/hmg/ddl156, 16782802

[ref67] Tarif A. M. M. Islam M. N. Jahan M. R. Afrin M. Meher M. M. Nozaki K. . (2023). Neurochemical phenotypes of huntingtin-associated protein 1 in reference to secretomotor and vasodilator neurons in the submucosal plexuses of rodent small intestine. Neurosci. Res. 191, 13–27. doi: 10.1016/j.neures.2022.12.023, 36581175

[ref68] Tarif A. M. M. Islam M. N. Jahan M. R. Yanai A. Nozaki K. Masumoto K. H. . (2021). Immunohistochemical expression and neurochemical phenotypes of huntingtin-associated protein 1 in the myenteric plexus of mouse gastrointestinal tract. Cell Tissue Res. 386, 533–558. doi: 10.1007/s00441-021-03542-4, 34665322

[ref69] Wroblewski G. Islam M. N. Yanai A. Jahan M. R. Masumoto K. H. Shinoda K. (2018). Distribution of HAP1-immunoreactive cells in the retrosplenial-retrohippocampal area of adult rat brain and its application to a refined neuroanatomical understanding of the region. Neuroscience 394, 109–126. doi: 10.1016/j.neuroscience.2018.10.029, 30367943

[ref70] Wu Y. Wang Y. Lu Y. Yan J. Zhao H. Yang R. . (2024). Research advances in huntingtin-associated protein 1 and its application prospects in diseases. Front. Neurosci. 18:1402996. doi: 10.3389/fnins.2024.1402996, 38975245 PMC11224548

[ref71] Yanai A. Islam M. N. Hayashi-Okada M. Jahan M. R. Tarif A. M. M. Nozaki K. . (2020). Immunohistochemical relationships of huntingtin-associated protein 1 with enteroendocrine cells in the pyloric mucosa of the rat stomach. Acta Histochem. 122:151650. doi: 10.1016/j.acthis.2020.151650, 33161374

[ref72] Yuen E. Y. Wei J. Zhong P. Yan Z. (2012). Disrupted GABAAR trafficking and synaptic inhibition in a mouse model of Huntington's disease. Neurobiol. Dis. 46, 497–502. doi: 10.1016/j.nbd.2012.02.015, 22402331 PMC3323696

[ref73] Zhao Z. Wang L. Gao W. Hu F. Zhang J. Ren Y. . (2017). A central Catecholaminergic circuit controls blood glucose levels during stress. Neuron 95, 138–152.e5. doi: 10.1016/j.neuron.2017.05.031, 28625488

