# Supplementary material for: Cell-type-specific localization of huntingtin-associated protein 1 in neuroendocrine and neurosecretory systems of the mouse hypothalamic–pituitary axis
Source: Front Cell Neurosci. 2026 Oct 26;20:1963485. doi: 10.3389/fncel.2026.1963485 (PMC13745205; doi:10.3389/fncel.2026.1963485)
Supplement: Supplementary file 1 [file Data_Sheet_1.PDF]

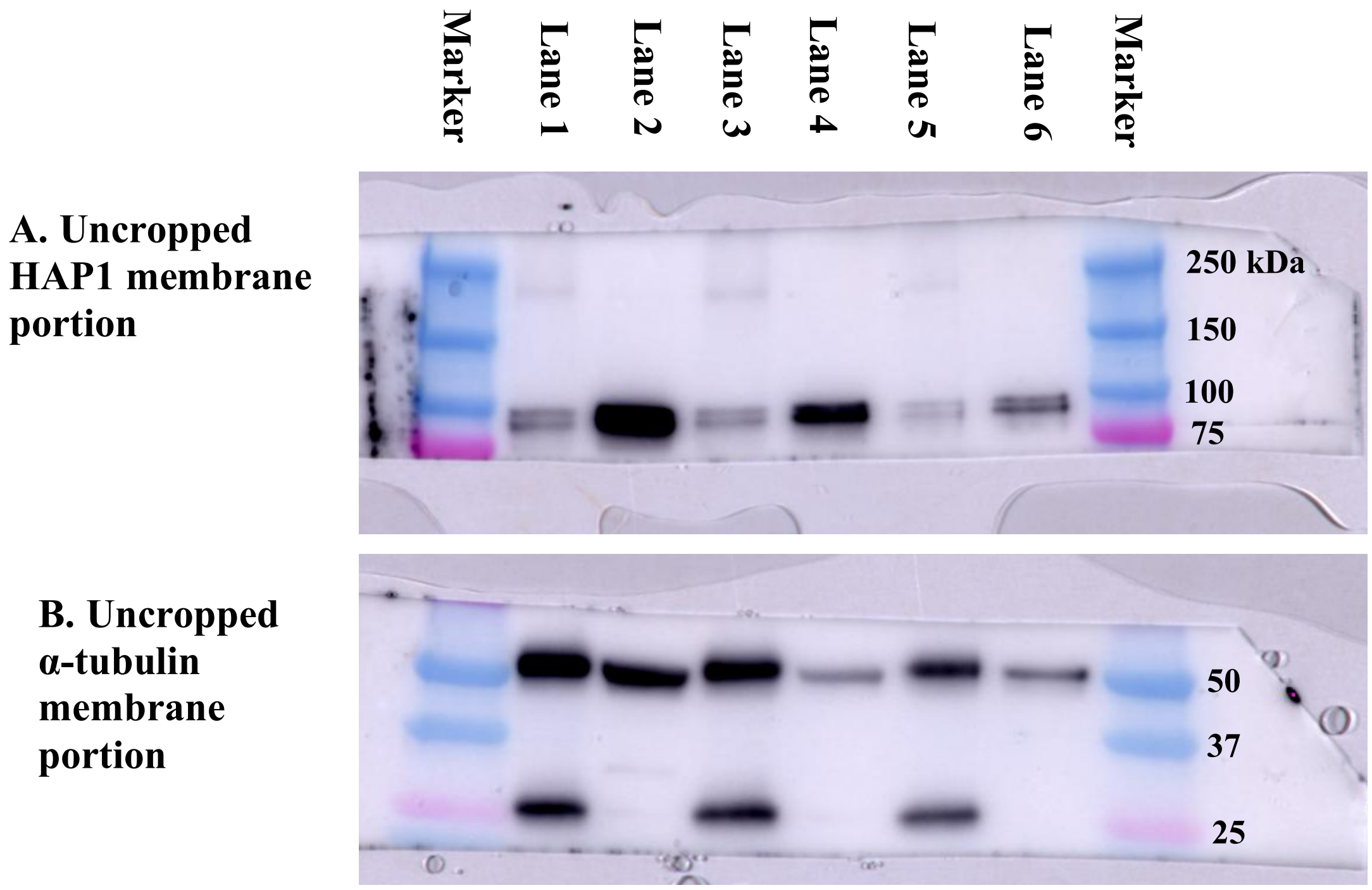

Protein loaded	Pituitary	Hypothalamus
20 µg	Lane 1	Lane 2
10 µg	Lane 3	Lane 4
5 µg	Lane 5	Lane 6

Supplementary Fig. S1. Uncropped Western blot membrane images corresponding to Fig. 1A. Uncropped original membrane images showing HAP1 (A) and α-tubulin (B) immunoreactivity in mouse pituitary and hypothalamic tissue lysates. Following electrophoretic transfer, the same membrane was horizontally divided according to molecular-weight range. The upper membrane portion was processed for HAP1 immunodetection, whereas the lower portion was processed separately for α-tubulin detection. Pituitary and hypothalamic lysates were examined at three protein-loading amounts (20, 10, and 5 µg per lane). Molecular-weight marker lanes (Precision Plus Protein™ Dual Color Standards, Bio-Rad Laboratories, catalog no. 161-0374) are shown in the original images. The pituitary (lane 1) and hypothalamic (lane 2) samples, each containing 20 µg protein, were used for the representative Western blot shown in Fig. 1A. HAP1, huntingtin-associated protein 1.
